# A gp41 MPER-specific Llama VHH Requires a Hydrophobic CDR3 for Neutralization but not for Antigen Recognition

**DOI:** 10.1371/journal.ppat.1003202

**Published:** 2013-03-07

**Authors:** David Lutje Hulsik, Ying-ying Liu, Nika M. Strokappe, Simone Battella, Mohamed El Khattabi, Laura E. McCoy, Charles Sabin, Andreas Hinz, Miriam Hock, Pauline Macheboeuf, Alexandre M. J. J. Bonvin, Johannes P. M. Langedijk, David Davis, Anna Forsman Quigley, Marlén M. I. Aasa-Chapman, Michael S. Seaman, Alejandra Ramos, Pascal Poignard, Adrien Favier, Jean-Pierre Simorre, Robin A. Weiss, C. Theo Verrips, Winfried Weissenhorn, Lucy Rutten

**Affiliations:** 1 Unit of Virus Host Cell Interactions (UVHCI), UMI 3265, Université Joseph Fourier-EMBL-CNRS, Grenoble, France; 2 Biomolecular Imaging (BMI), Faculty of Science, Utrecht University, Utrecht, The Netherlands; 3 MRC/UCL Centre for Medical Molecular Virology, Division of Infection and Immunity, University College London, London, United Kingdom; 4 Bijvoet Center for Biomolecular Research, Faculty of Science, Utrecht University, Utrecht, The Netherlands; 5 Pepscan Therapeutics BV, Lelystad, The Netherlands; 6 Department of Virology, Biomedical Primate Research Centre (BPRC), Rijswijk, The Netherlands; 7 Division of Viral Pathogenesis, Beth Israel Deaconess Medical Center, Harvard Medical School, Boston, Massachusetts, United States of America; 8 Department of Immunology and Microbial Science, International AIDS Vaccine Initiative Neutralizing Antibody Center, The Scripps Research Institute, La Jolla, California, United States of America; 9 International AIDS Vaccine Initiative, New York, New York, United States of America; 10 CNRS, Institut de Biologie Structurale-Jean-Pierre Ebel, Grenoble Cedex, France; 11 CEA, Institut de Biologie Structurale-Jean-Pierre Ebel, Grenoble Cedex, France; 12 UJF-Grenoble-1, Institut de Biologie Structurale-Jean-Pierre Ebel, Grenoble Cedex, France; 13 QVQ BV, Utrecht, The Netherlands; Harvard University, United States of America

## Abstract

The membrane proximal external region (MPER) of the HIV-1 glycoprotein gp41 is targeted by the broadly neutralizing antibodies 2F5 and 4E10. To date, no immunization regimen in animals or humans has produced HIV-1 neutralizing MPER-specific antibodies. We immunized llamas with gp41-MPER proteoliposomes and selected a MPER-specific single chain antibody (VHH), 2H10, whose epitope overlaps with that of mAb 2F5. Bi-2H10, a bivalent form of 2H10, which displayed an approximately 20-fold increased affinity compared to the monovalent 2H10, neutralized various sensitive and resistant HIV-1 strains, as well as SHIV strains in TZM-bl cells. X-ray and NMR analyses combined with mutagenesis and modeling revealed that 2H10 recognizes its gp41 epitope in a helical conformation. Notably, tryptophan 100 at the tip of the long CDR3 is not required for gp41 interaction but essential for neutralization. Thus bi-2H10 is an anti-MPER antibody generated by immunization that requires hydrophobic CDR3 determinants in addition to epitope recognition for neutralization similar to the mode of neutralization employed by mAbs 2F5 and 4E10.

## Author Summary

Due to the absence of an effective vaccine or cure for acquired immunodeficiency syndrome (AIDS), HIV-1 infections still result in high mortality. Two antibodies, 2F5 and 4E10, previously isolated from HIV-1 infected patients, prevent infections by binding to the MPER of gp41, a part of the virus that is difficult to access and only transiently exposed. Here, we immunized llamas with a gp41-based immunogen and subsequently isolated a small antibody fragment (VHH) that can easily access and recognize the MPER. We showed that a unit of two VHH, named bi-2H10, was indeed capable of preventing HIV-1 from infecting cells. We determined the three dimensional structure of the VHH and mapped its interaction site to an MPER region that overlaps with the 2F5 epitope. The 2H10 VHH displays a membrane binding component important for neutralization that resembles that of 2F5. In conclusion, we have developed an immunogen and a small antibody that may have great potential for development of novel anti-HIV/AIDS vaccines and treatments.

## Introduction

The trimeric HIV-1 envelope glycoprotein (Env), composed of its receptor binding subunit gp120 and the fusion protein gp41, is the main target for neutralizing antibodies. Although recent studies have demonstrated the potential of the human immune system to produce broadly neutralizing antibodies (bnAbs) directed against gp120 [1]–[10], generation of antibodies with broad cross-clade neutralization activity via recombinant Env immunization has been rare [11]–[14]. This may be due in part to the long time frame required to generate such antibodies as well as to multiple evasive strategies developed by the virus [15]–[17].

Because Env gp41 contains highly conserved sequences that are exposed during the conformational changes leading to membrane fusion [18], [19] a considerable effort is underway to target gp41 with a focus on the membrane proximal external region (MPER). The MPER is recognized by the broadly neutralizing antibodies (bnAbs) 2F5, Z13, 4E10 and 10E8 [9], [20]–[23]. They interact with linear epitopes of the MPER [9], [24]–[26] and gp41-mAb interaction most likely prevents refolding of gp41 into the six-helical bundle conformation [27]–[29]. Notably, 2F5 and 4E10 are among the broadest cross-reactive human neutralizing antibodies directed against HIV-1 gp41 while recently-described 10E8 combines this extensive breadth with substantially increased potency [9], [22]. The potencies of 2F5 and 4E10 are confirmed by their ability to prevent HIV-1 transmission in rhesus macaques by passive immunization [30]–[35].

Numerous studies have been performed with purified gp41 proteins and gp41-derived peptides in an attempt to induce such antibodies by immunization; however, with very limited success so far [36]–[45]. This was partly attributed to the fact that both 4E10 and under some experimental conditions also 2F5 display lipid binding and potential polyreactivity [46], which may be a special feature of anti-HIV antibodies [47]. However mAb 10E8 does not bind lipids and is not polyreactive [9]. 2F5 and 4E10 contain hydrophobic residues within the third complementarity-determining region of the heavy chain (CDR H3), which do not contact the antigen directly, but are required for virus neutralization [48]–[51]. CDR H3 was suggested to insert into the viral membrane and extract membrane-embedded MPER leading to tight binding [52], [53]. In addition CDR H3 of 2F5 may function in destabilizing the helical region downstream of the core 2F5 epitope leading to the extended-loop conformation of the 2F5 epitope [54]. Both models are consistent with the finding that neutralization activity of MPER antibodies depends on the transmembrane region allowing functional MPER exposure [55]. Furthermore, MPER antibody epitopes of most primary isolates become only accessible after receptor/co-receptor-binding induced conformational changes in Env [56], [57]. In addition, MPER mAb Env recognition was reported to induce gp120 shedding leading to irreversible neutralization effects [58]. Moreover, MPER antibody epitope access may be size-restricted [59], which is consistent with the proposal that such antibodies target the transient fusion-intermediate conformational state of gp41 [60] that is also difficult to access by HR1-specific neutralizing antibodies [61]–[63]. Accordingly, anti-MPER-like neutralization activity in patient sera was reported to be rare [64]–[67] or absent [68], [69]. This was attributed to the fact that MPER-specific antibodies in acutely infected individuals are polyreactive [70] and their B cells may undergo clonal deletion [71]. However, a recent study suggested that 10E8-like potent anti-MPER antibodies occur frequently in sera with high neutralizing breadth and potency without exhibiting autoreactivity such as lipid interaction [9].

Thus a major challenge remains to induce MPER-specific neutralizing antibodies upon immunization. Therefore we immunized two llamas with proteoliposomes containing trimeric gp41 consisting of the HIV-1 Env transmembrane region and MPER. Llamas (*Lama glama*) and other *Camelidae* produce classical antibodies as well as heavy chain-only antibodies [72]. Notably, VHH (variable domain of the heavy chain of a heavy chain only antibody) specific for gp120 with broad neutralizing activity have been successfully produced by immunization before [73]–[78]. Here we report the functional and structural characterization of the llama anti-MPER VHH 2H10. Our data suggest that this is the first MPER-specific antibody induced by immunization that employs similar structural features as mAbs 2F5 and 4E10 for neutralization. 2H10 requires a hydrophobic CDR3 for neutralization but not for interaction with its epitope, like mAbs 2F5 and 4E10. Futhermore, 2H10 binds to a 2F5 overlapping epitope that is in an alpha-helical conformation, which provides important insights for the development of MPER based vaccines.

## Materials and Methods

### Ethics statement

This study was carried out in strict accordance with the Dutch Experiments on Animals Act 1997. In accordance with article 18 of the Act the protocol was assessed and approved by the Animal Ethics Committee of the Utrecht University (Permit Number: DEC#2007.III.01.013). All efforts were made to minimize discomfort related to immunizations and blood sampling. The animal welfare officers of the Utrecht University checked the mandatory administration and supervised the procedures and the well-being of the llamas that were used.

### Preparation of gp41 constructs

The HIV-1 gp41 constructs ‘gp41CHRTM’, clade B C-terminal Heptad Repeat (CHR), MPER and transmembrane (TM) regions (residues 629–706, HXB2c numbering), ‘gp41-GCN’, gp41 residues 629–683 (HXB2c numbering) fused to a trimeric GCN4 leucine zipper [79], and ‘gp41_INT_’, which has a similar design as reported [60] with the exception that the trimeric GCN4 zipper was fused in frame with part of gp41 NHR, were cloned into pETM-11 (EMBL, Heidelberg) or pETM-13 using standard PCR techniques. Proteins were expressed in *Escherichia coli* strains C41 or BL21. Cells were grown to an OD_600_ of 0.8 and induced with 1 mM isopropyl-ß-D-thiogalactopyranoside (IPTG). Cells were harvested by centrifugation and lysed in either phosphate buffered saline (PBS) supplemented with 1% 3-[(3-cholamidopropyl)dimethylammonio]-1-propanesulfonate (CHAPS) or lysed in 20 mM tris(hydroxymethyl)aminomethane hydrogen chloride (Tris-HCl) pH 8.0, 100 mM NaCl, or in 20 mM Bicine pH 9.3, 100 mM NaCl, for ‘gp41CHRTM’, ‘gp41-GCN’ and ‘gp41_INT_’ respectively. Purification was performed using a Ni^2+^ affinity or Q-sepharose column (‘gp41_INT_’) and gel filtration on a Superdex 200 column (GE Healthcare). Gp41_528–683_ was produced as described [29].

### Preparation of immunogen

The concentration of gp41CHRTM was adjusted to 1 mg/ml. Lipids POPC, POPE, POPS, Cardiolipin/Sphingomyelin and cholesterol (Avanti Polar Lipids) were dissolved in chloroform and mixed in a 1∶2∶1∶2∶5 ratio (w/w). After solvent evaporation, the lipid film was dried under vacuum. Multilamellar vesicles were obtained by resuspending the lipid film in PBS to a final concentration of 1 mg/ml. After three quick freeze/thaw rounds the liposome suspension was extruded through a 100 nm membrane. Liposomes and gp41CHRTM were mixed in a 1∶1 ratio (w/w). CHAPS was removed by extensive dialysis with PBS containing 0.1 mg/ml BioBeads (BioRad), changing the solution every eight hours for two days. The quality of gp41CHRTM proteoliposomes was confirmed by dynamic light scattering (Malvern Nano ZS).

### Immunization and construction of phage libraries

Two llamas (L6 and L7) were immunized intramuscularly with the gp41CHRTM reconstituted in the trimeric form into synthetic liposomes (gp41 proteoliposomes) without adjuvant. The immunization scheme consisted of a priming immunization at day 0 followed by 5 boosts, which were given at days 7, 14, 21, 28 and 35. During the prime and the first boost 0.25 mg of gp41 and 0.5 mg of lipids were used per injection. These amounts were halved for the remaining boosts. Importantly no adjuvants were used. The immune response was measured in the serum taken at day 21 and compared to day 0. At day 43 lymphocytes were collected from the two llamas for construction of two separate phage libraries, as described previously [80]. The genes encoding for the VHH were amplified and cloned in the phage-display plasmid, pAX50. The plasmids were transferred to *E. coli* strain TG1 [supE hsdΔ5 thi Δ(lac-proAB) F′(traD36 proAB+,lacIq lacZΔM15)].

### Selection of gp41-specific VHH

Two rounds of selection with phage libraries were carried out by panning phages onto gp41-proteoliposomes immobilized in microtiter plate wells. In the first round selection, gp41 proteoliposomes at 5, 1 and 0.2 µg were coated in the wells of PolySorp (Nunc) microplate overnight at 4°C followed by PBS containing 4% Marvel dried skimmed milk (Premier International Foods UK Ltd) for one hour at room temperature. approximately 10^10^ of the phages, pre-incubated for 30 minutes in 2% skimmed milk in PBS and empty liposomes to reduce nonspecific binding, were then added to the wells for 2 hours followed by extensive washing (15 times) with PBS containing 0.05% Tween 20 (PBS-T)(the 5^th^, 10^th^ and 15^th^ wash steps were done for 10 min) and three times with PBS. The bound phages were eluted with 100 mM TEA, neutralized with 1 M Tris-HCl pH 7.5, and rescued by infection of the *E. coli* strain TG1, which were then plated on LB agar plates containing 100 µg/ml ampicillin and 2% glucose. The phages eluted from 1 µg coated gp41 proteoliposomes in the first round yielded the highest enrichment of ∼100 times. These were therefore chosen for further selection in a second round. The selected polyclonal phages were amplified in *E. coli* strain TG1 after infection with helper phages VCSM13 (Stratagene). After purification from the culture supernatant, phages were used as input for the second round selection. Lower concentrations of proteoliposomes (0.5 and 0.1 µg per well) were coated. ∼10^9^ phages of the amplified phages were incubated in each well. After extensively washing away unbound phages, the bound phages were eluted by 100 µl of 150 µg/ml human monoclonal antibodies of either 2F5 or 4E10. TG1 cells were infected with the eluted phages. The infected TG1 cells were grown on LB agar plates containing 100 µg/ml ampicillin and 2% glucose to obtain individual colonies. The infected TG1 were also spotted on the LB agar plates supplemented with 100 µg/ml ampicillin and 2% glucose for the assessment of the efficiency of the selection. The enrichment of specific phages was estimated in comparison with the phages that were eluted by an irrelevant human monoclonal antibody (Remicade, Schering-Plough) or with the phages eluted from the empty wells. DNA inserts of a number of selected phages binding to gp41 were recloned to plasmid pAX51 using the restriction sites *Sfi*I and *Bst*EII.

### Phage ELISA

MaxiSorp (Nunc) plate wells were coated overnight at 4°C, with gp41-GCN in PBS. After blocking of the wells with 4% skimmed milk in PBS, they were incubated with VHH fused to M13 phages in the presence of 2% skimmed milk. The wells were washed with PBS-T, and bound phages were detected by incubation with a mouse monoclonal antibody against M13 phage coupled to horse radish peroxidase (HRP). The amount of HRP was visualized by the addition of O-phenylenediamine (OPD) supplemented with 0.03% H_2_O_2_. The reactions were stopped by the addition of 1 M H_2_SO_4_ and the optical density was measured at 490 nm.

### Production and purification of gp41-specific VHH

TG1 strains expressing monoclonal VHH were cultivated in 2×YT medium supplemented with 100 µg/ml of ampicillin until the optical density of culture medium reached 0.5 at 600 nm. Then 1 mM IPTG was added to induce VHH production for 5 hours at 37°C. VHH were extracted from the periplasmic fraction and bound to Talon metal-affinity resin (Clontech 635504). After washing away unbound material, the bound VHH were eluted by 300 mM imidazole in PBS. The eluted VHH were dialyzed against PBS with two buffer changes. The VHH were dialyzed twice for 1.5 hours at room temperature, followed by dialysis overnight at 4°C. The VHH were analyzed on 15% acrylamide sodium dodecyl sulfate polyacrylamide gel electrophoresis (SDS-PAGE) stained by Coomassie Brilliant Blue and a Western blot using the antibody against the myc-tag (9E10).

### Binding of VHH to HIV-1 envelope proteins in ELISA

The binding of the VHH to HIV-1 envelope was performed on the immobilized gp41 and gp140-92UG037 in the microtiter plate (MaxiSorp from Nunc) in ELISA. 200 ng Gp41-GCN or 280 ng gp140-92UG037 protein were coated in the wells of microtiter plates overnight at 4°C followed by incubation with 4% skimmed milk to block non-specific binding. Dilution series of purified VHH were incubated with the coated wells for one hour at room temperature followed by the removal of unbound VHH by washing three times with PBS-T and one time with PBS. Bound VHH were detected with anti-myc tag antibody (9E10) and donkey anti mouse peroxidase (DAMPO). The antibodies were incubated for one hour and washed with PBS-T and PBS between each incubation. The plates were developed using OPD containing 0.03% H_2_O_2_. The reaction was stopped with 1 M H_2_SO_4_. The optical density was measured at 490 nm.

### Binding of VHH to membrane anchored Env

Titrating amounts of antibodies were added to JR-FL HIV-1 Env-transfected 293T cells expressing either cleaved or uncleaved Env, incubated for 1 h at 37°C and washed with FACS buffer (PBS, 5% heat-inactivated fetal calf serum [HIFBS], 0.02% azide). MAbs b6, b12, and 4E10 were detected with a secondary anti-IgG Ab conjugated to phycoerythin (Jackson ImmunoResearch). MAb b12 and the VHH 2H10, bi-2H10 and bi-2H10 W100A were biotinylated and detected with streptavidin PE. MAb b6 binding is weak on cleaved Env, confirming that most Env is cleaved [81]. Binding was analyzed using flow cytometry, and binding curves were generated by plotting the mean fluorescence intensity of antigen binding as a function of antibody concentration.

### Bivalent VHH construction

PCR was used to amplify the VHH sequences. Different primer sets designed to amplify the VHH that will be located at the N terminus and the VHH that will be located at the C terminus of the bivalent VHH. The primers at the 3′ of the N-terminal VHH and at the 5′ of the C-terminal VHH, may encode a flexible sequence glycine-serine (GS) linker represented by a repeat of the pentapeptide “G-G-G-G-S”, also called 5GS. These same primers contain a unique restriction site (*Bam*HI). We constructed bi-2H10 with 5GS, 15GS, and 25GS linkers and with the linker GGGGSGGGGSGGGGGGS, called 17GS. For the studies we used the bi-2H10 with the 17GS linker, unless specified differently. After PCR amplification, the generated fragments were cleaved at a unique N-terminal restriction site (*Sfi*I restriction site) and *Bam*HI for the VHH that will be located at the N terminus, and with *Bam*HI and at a unique C-terminal restriction site (*Bst*EII) for VHH that will be located at the C terminus. The fragments are ligated into pAX51, which was digested with *Sfi*I and *Bst*EII.

### Lipid binding assays

Antibodies were mixed with liposomes having the lipid composition as described above in PBS and their buffer was adjusted to 40% sucrose. The 40% sucrose mixture was overlaid stepwise with 30, 20, 10 and 5% sucrose and centrifuged in a Ti55 swinging bucket rotor for 18 h at 50 K rpm. Six fractions were recovered from the gradient and samples thereof were analyzed by SDS-PAGE.

For ELISA analysis, cardiolipin from bovine heart (SIGMA, C0563) and l-α-phosphatidyl-l-serine from bovine brain (SIGMA, P7769) were dissolved in ethanol to a concentration of 5 mg/ml and further diluted with PBS to 50 µg/ml of which 100 µl was coated per well in PolySorp plates (Nunc) overnight at room temperature without a cover foil to allow complete evaporation of the sample. As a control gp140-92UG037 was coated on a MaxiSorp plate (Nunc). Dilution series were prepared of 2H10, bi-2H10, 2F5 and 4E10. 2F5 and 4E10 were detected with the mouse anti-human IgG monoclonal 3E8 (SANTA CRUZ). The rest of the ELISA was performed as described above.

### Viruses

HIV-1 IIIB was obtained from the Centralised Facility for AIDS Reagents (CFAR), NIBSC and propagated in H9 cells. Replication-competent NL43 stocks were prepared from a replication-competent HIV-1 molecular clone by transfection of 293T cells. All other used HIV-1 viruses were HIV-1 envelope pseudotyped viruses and were produced in 293T cells by co-transfection with the pSG3Δ*env* plasmid and the relevant envelope plasmid. The subtype B HIV-1 reference panel of envelope clones and the subtype C clones were obtained through the NIH AIDS Research and Reference Reagent Program, Division of AIDS, NIAID, NIH, USA. AC10.0 clone 29 and SC422661 clone 8 were contributed by David Montefiori, Feng Gao and Ming Li; ZM109F.PB4 was contributed by David Montefiori, Feng Gao, S Abdool Karim and G Ramjee; RHPA4259 clone 7 was contributed by B.H. Hahn and J.F. Salazar-Gonzalez. The SHIV clones were constructed at the BPRC by Zahra Fagrouch and Ernst Verschoor. The panel of transmitted subtype B (T/F) clones (WEAU-d15.410.787, 1006-11.C3.1601, 1054-07.TC4.1499, 1056-10.TA11.1826, 1012-11.TC21.3257) were provided by George Shaw and Beatrice Hahn.

### Cells

TZM-bl cells were obtained through the NIH AIDS Research and Reference Reagent Program from J. C. Kappes, X. Wu, and Tranzyme, Inc., and cultured in Dulbecco's modified Eagle medium (Invitrogen) containing 10% (v/v) fetal calf serum (FCS).

### Neutralization assay

Neutralization was measured using pseudovirus or 200 50% tissue culture infective doses (TCID50) of virus in the TZM-bl cells as targets following the methods of Derdeyn et al. [82] Wei et al. [83] and Li et al. [84], with Bright-Glo luciferase reagent (Promega, Southampton, UK). The neutralization activity of each VHH was assayed in duplicate. No virus inactivation was observed with a negative control VHH. VHH IC50 titers were calculated using the XLFit4 software (ID Business Solutions, Guildford, UK). The HIV-1 neutralization potency of llama 6 & 7 serum/plasma samples were also evaluated in TZM-bl. Neutralization was measured using 200 TCID50 of IIIB virus in the TZM-bl cell-based assay, as described above, using a Glomax plate reader (Promega). Serum samples were heat-inactivated to inactivate complement by incubation at 56°C for 1 h before use in neutralization assays. Threefold serial dilutions of llama sera were then tested, starting at a 1∶5 dilution.

### Epitope mapping

The binding of VHH and mAb to peptides was assessed in a Pepscan-based ELISA [85]. Structural aspects of antibody antigen interaction were revealed through small random peptide libraries [86]. Each VHH and mAb was titrated to ensure that optimal binding was achieved and that nonspecific binding was avoided. Each well in the card contained covalently linked peptides that were incubated overnight at 4°C with VHH and mAb, between 1 and 10 ng/ml in PBS containing 5% horse serum (v/v), 5% OVA (w/v), and 1% (v/v) Tween 80, or in an alternative blocking buffer of PBS containing 4% horse serum (v/v), and 1% (v/v) Tween 80. After washing, the plates were incubated with a HRP-linked rabbit anti-Ab (DakoCytomation) for 1 h at 25°C. After further washing, peroxidase activity was assessed using substrate (0.5 g/l 2,2′-azino-di[3-ethyl-benzthiazolinesulfonate(6)]diammonium salt (ABTS)) with 0.006% H_2_O_2_ in 0.18 M Na_2_HPO_4_, 0.22 M citric acid was added until the solution had a pH of 4. The color development was quantified after 60 minutes using a charge-coupled device camera and an image-processing system.

### Protein expression and purification of 2H10 for crystallization

The cDNA corresponding to 2H10 was synthesized (GeneArt) and cloned into vector pMEK220 (without 6his-tag or myc tag) and 2H10 was expressed in *E. coli* strain BL21gold (DE3) (Invitrogen). Cells were grown to an OD600 of 0.7 and induced with 1 mM IPTG at 37°C. After 5 hours cells were harvested by centrifugation and lysed by sonication in 0.02 M 4-(2-hydroxyethyl)-1-piperazineethanesulfonic acid (HEPES), 0.1 M NaCl, pH 7.4 (HEPES buffer). 2H10 was purified by affinity chromatography using a Protein A column (Roche). 2H10 was eluted with 0.01 M glycine, pH 2.5, and fractions containing 2H10 were neutralized with 1 M Tris-HCl, pH 8. These fractions were further purified by gel filtration on a Superdex 200 column (GE Healthcare) in HEPES buffer. Purity of the sample was confirmed on a 12% SDS-Tris-Tricine gel.

### Crystallization, data collection and structure determination

2H10 in complex with peptide 903 was subjected to crystallization at a concentration of 12 mg/ml. Crystals were obtained by the vapor diffusion method in hanging drops, with equal volumes of antibody and reservoir solution (0.1 M Bicine, 3.2 M NH_4_SO_4_, pH 9). The crystal was cryo-cooled at 100 K in reservoir buffer containing 25% (v/v) glycerol. A complete dataset was collected at the ESRF (Grenoble, France) beamline ID14-4. Data were processed and scaled with MOSFLM [87], and SCALA [88]. The crystals belong to space group I23 with unit cell dimensions of a = 89.95 Å, b = 89.95 Å, c = 89.95 Å and α = 90.00°. The structure was solved by molecular replacement using PHASER [89] and the VHH structure from Protein Data Bank (PDB) ID 3EZJ as a search model. An initial model was built with ARP/wARP [90] and completed by several cycles of manual model building with Coot [91] and refinement with refmac [92] using data to 1.3 Å resolution. The final model contains 2H10 residues 1–122. Molecular graphics figures were generated with PyMOL (W. Delano; http://www.pymol.org). Coordinates and structure factures have been deposited in the Protein Data Bank with accession ID 4B50.

### NMR analyses of 2H10 and 2H10 in complex with its peptide epitope

A ^13^C,^15^N-labeled protein sample was prepared at a concentration of 0.95 mM in 20 mM HEPES buffer and 75 mM NaCl at pH 6.7 with 10% D_2_O. For the study of the peptide∶2H10 complex, a solution of gp41 peptide (_655_KNEQELLELDKWASL_669_.) was prepared at 7.9 mM in the same HEPES buffer and 5 µl were added step by step to the ^13^C,^15^N-labeled protein sample. A final protein∶peptide ratio of 1∶1.5 was obtained and ^15^N HSQC experiments were recorded for each peptide addition in order to control the complete disappearance of the free form. Sequential backbone resonance assignments of free and peptide bound 2H10 were performed by recording series of 3D experiments using the BEST scheme (HNCO, HNCA, HNcoCACB, HNCACB) and the BEST-TROSY scheme (hNcocaNH, hNcocaNH) [93].

All NMR experiments were performed at 37°C on Agilent VNMRS 600 or 800 MHz spectrometers equipped with triple-resonance (^1^H, ^13^C, and ^15^N) coldprobes and shielded z-gradients. NMR data were processed with NMRPipe [94] and analyzed using the CcpNmr Analysis routines of CCPN [95]. Chemical shifts were referenced with respect to the H_2_O signal at 4.66 ppm relative to DSS, using the ^1^H∶X frequency ratios of the zero point according to Markley et al. [96].

### Surface plasmon resonance

Surface plasmon resonance (SPR) analysis was performed with a Biacore T100 (GE Healthcare). As a flow buffer 10 mM HEPES, 150 mM NaCl, pH 7.4 with 0.005% Tween-20 was used. Gp140-92UG037 was immobilized to 1500 response units using 50 µg/ml protein in flow buffer on an activated CM-5 sensor chip (GE Healthcare: BR-1000-50) according to the manufacturer's instructions. Specific binding to the target protein was corrected for nonspecific binding to the deactivated control channel. The flow rate was 30 µL/min. Regeneration of the sensor chip was achieved with 4 M MgCl_2_ for 60 seconds at 50 µL/min. Single concentration injections were all performed with 100 nM of VHH for 60 seconds each. All data were measured in duplo and the curves superpose quite well. Data were analyzed with the Biacore T100 software version 2. Curves obtained for 2H10 could not be fitted well with the 1∶1 Langmuir or heterogeneous ligand algorithms, but only with the two-state reaction algorithm. To establish whether 2H10 indeed binds with a two-state reaction, we injected 2H10 with different injection durations. This clearly showed that the dissociation rate is dependent on the injection time. The longer the duration of the injection, the stronger the interaction becomes and the dissociation rate decreases. We, therefore, fitted the curves belonging to the monovalent 2H10 VHH with the two-state reaction model. Curves of the bivalent 2H10 should be fitted with a combination of two-state reaction and bivalent analyte model, but due to a lack of availability of this model, the curves were fitted with the heterogeneous ligand fit and the two-state reaction fit.

Additional SPR analysis was performed using a Biacore 3000 (GE Healtcare). As a flow buffer 10 mM HEPES, 150 mM NaCl, pH 7.4, 50 µM EDTA with 0.005% P-20 (GE Healthcare) was used. Gp41_INT_ or gp41_528–683_ was immobilized to 1000 response units in a sodium acetate buffer, pH 4.5, on an activated CM-5 sensor chip according to the manufacturer's instructions. Specific binding to the target protein was corrected for nonspecific binding to the deactivated control channel. The flow rate was 20 µl/min. Regeneration of the sensor chip was achieved with 25 mM NaOH for 30 seconds at 50 µl/min. Data were analyzed with the BIAevalution software version 4.1. The curves for 2H10 and 2H10-W100A on gp41_INT_ were fitted with separate association and dissociation, because global fittings, either 1∶1 langmuir or two-state reaction, did result in reasonable Chi^2^ scores and residuals.

### Isothermal titration calorimetry

Isothermal Titration Calorimetry (ITC) measurements were carried out at 25°C on a VP-ITC microcalorimeter (MicroCal). Both 2H10 and peptides were purified or dissolved, respectively, in a solution containing 20 mM HEPES, pH 7.4 and 100 mM NaCl. The samples were degassed for 20 min and centrifuged to remove any residuals prior to the measurements. The concentration of 2H10 in the sample cell was 10 µM and that of peptide 903 or 904 in the syringe was approximately 100 µM. The peptides injected into buffer alone was used as a negative control. Data were fit to a single binding site model and analyzed using Origin version 5 (MicroCal) software.

### Site directed mutagenesis

Site directed mutagenesis was performed by PCR with complementary oligos which carried the desired mutation, to amplify the whole plasmid. The oligo's were designed to be between 42–50 nucleotides long and have a melting temperature (salt adjusted, calculated on http://www.basic.northwestern.edu/biotools/oligocalc.html) of approximately 80°C. The PCR reaction was performed with 20 ng of template DNA (2H10-wild type plasmid), 125 ng each primers, 2 mM DNTPs, Pfu buffer with 2 mM, MgSO_4_ and 0.5 units of Pfu polymerase (Fermentas). The protocol consisted of an initial denaturation step at 95°C for 5 min followed by 16 cycles of 95°C for 30 seconds, 60°C for 1 minute and 68°C for 7 minutes, and a final step extension at 68°C for 10 min. DNA was cleaned with PCR Clean-up kit (Macherey-Nagel). Parental methylated DNA was digested with DpnI (Fermentas), followed by transformation into TG1 *E. coli* cells.

### Modeling of the gp41 peptide interaction with 2H10

Models of gp41 peptide docked onto 2H10 were built with the webserver version “The HADDOCK web server for data-driven biomolecular docking” of HADDOCK2.1 [97] using CNS1.2 [98] for the structure calculations. The coordinates of 2H10 were taken from the crystal structure, and the gp41 peptide coordinates were based on those of the helical peptide (653-QEKNEQELLELDKWASL-669) from the crystal structure of a late fusion intermediate of HIV-1 gp41 (PDB code: 2X7R [29]). Ambiguous interaction restraints were defined, based on the NMR chemical shift perturbation (CSP) mapping using the program SAMPLEX [99] and the epitope mapping. The SAMPLEX program takes as input a set of data and the corresponding three-dimensional structure and returns the confidence for each residue to be in a perturbed or unperturbed state. Residues 47–51, 57–62 and 95–99 identified as perturbed in 2H10 upon binding to gp41 and residues 657, 658, 661, 662 and 665 identified from the epitope mapping, were used as interaction restraints for docking. Phi-psi angles were deduced from the ^13^C chemical shifts using TALOS+ [100] and the resulting dihedral and H-bond restraints were used in the modeling as well. The docking was performed with default HADDOCK parameters, except that random removal of restraints was turned off and the clustering cutoff was decreased to 2.5 Å because of the small size of the peptide. Non-bonded interactions were calculated with the OPLS force field using a cutoff at 8.5 Å. The electrostatic potential (E_elec_) was calculated by means of a shift function, while a switching function – between 6.5 and 8.5 Å – was used for the Van der Waals potential (E_vdw_). The HADDOCK score is used to rank the generated models. It consists of a weighted sum of intermolecular electrostatics, Van der Waals, desolvation (ΔG_solv_) [101] and ambiguous interaction restraint (AIR) energies, defined as: *Haddock Score* = 0.2*E_elec_*+1.0*E_VdW_*+1.0*E_ΔGsolv_*+0.1*E_AIR_*

## Results

### Immunization of llamas, library construction and selection of VHH against gp41

**Figure 1:**
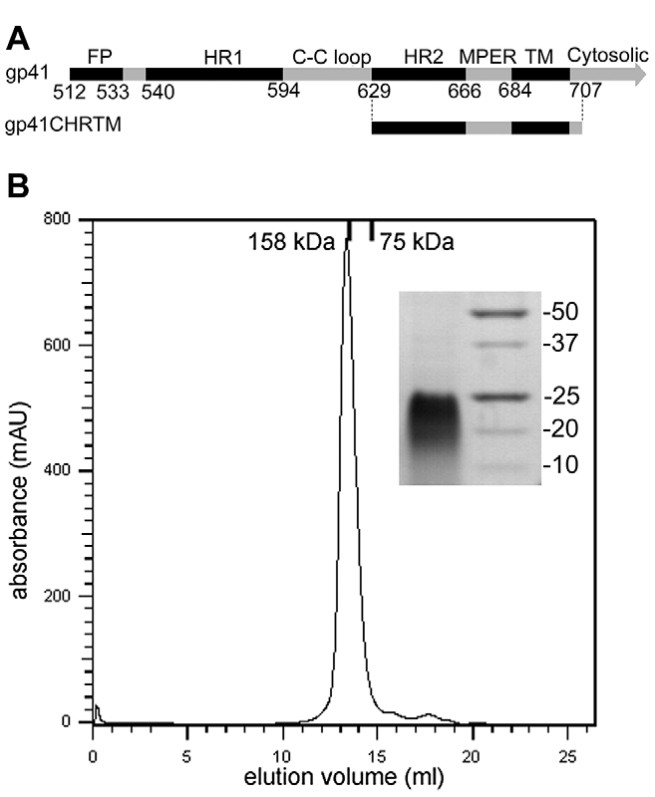
The gp41CHRTM antigen used for immunization. **A**) Schematic representation of gp41 and of the regions present in gp41CHRTM. FP, fusion peptide; HR1, N-terminal heptad repeat; C-C loop, cysteine loop, HR2, C-terminal heptad repeat; MPER, membrane proximal external region; TM, transmembrane region. The residue numbers at the domain/region boundaries are given. **B**) Gel filtration chromatogram of recombinant gp41CHRTM, which elutes at 13.3 ml from the column, similar to the elution profile of a marker protein of 158 kDa. This indicates that gp41CHRTM is most likely trimeric and may have an elongated structure. The inset shows a Coomassie stained SDS-PAGE gel with the gp41CHRTM protein band at the left and a protein marker at the right with the marker protein sizes in kDa indicated at the right.

**Figure 2:**
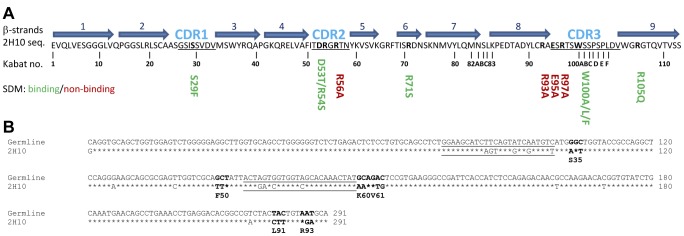
Amino acid and DNA sequence of 2H10. **A**) Amino acid sequence. Secondary structure assignment is based on the crystal structure. β-strands are depicted as blue arrows. CDR regions are indicated with CDR1 to CDR3 and are underlined. Residues depicted in bold were mutated. Mutants that still showed binding are shown in green and mutants that do not bind to the antigen anymore are shown in red. **B**) DNA sequence alignment of 2H10 with the germline V-gene from which it originated. The asterisks indicate identical nucleotides. The CDR1 and CDR2 coding regions are underlined. Codons with at least two point mutations are in boldface.

Two llamas (L6 and L7) were immunized with the gp41 proteoliposomes composed of an HIV-1-like lipid envelope [102] and membrane-anchored gp41CHRTM (Fig. 1). The presence of anti-gp41CHRTM proteoliposome antibodies in the plasma was confirmed by ELISA at day 21 post immunization. However, no significant neutralization was detected in the sera/plasma at day 21 and 43 compared to those of day 0. Two phage display libraries were constructed and the selection of VHH targeting gp41 was performed in two rounds by direct panning of the phage display library on immobilized gp41 proteoliposomes. In the first round triethyl-amine (TEA) was used to elute the phages. In the second round a specific competitive elution with bnAbs 2F5 or 4E10 was performed. Monoclonal VHH expressing TG1 clones from first and second round selections were screened for binding to detergent-solubilized gp41CHRTM and gp41-GCN (with pIIGCN in place of the transmembrane region) by phage ELISA. Approximately 80% of all monoclonal VHH displaying phages were positive for binding. In order to find VHH targeting specific binding sites, a competition ELISA between phages and bnAb 2F5 or 4E10 was performed. The phages with either strong binding signals, and/or binding signals that were inhibited by bnAb 2F5 or 4E10 were chosen for further investigation with purified VHH. Using ELISA, three of the VHH were found to bind to gp41-GCN, gp41 proteoliposomes and gp140-92UG037. 2H10, which originated from llama 7 (L7), exhibited the highest maximum signal and was therefore chosen for the subsequent studies (see Fig. 2 for the sequence of 2H10). 2H10 competed for binding to gp41 with 2F5, but not with 4E10.

### Epitope mapping of 2H10 with Pepscan analysis

**Figure 3:**
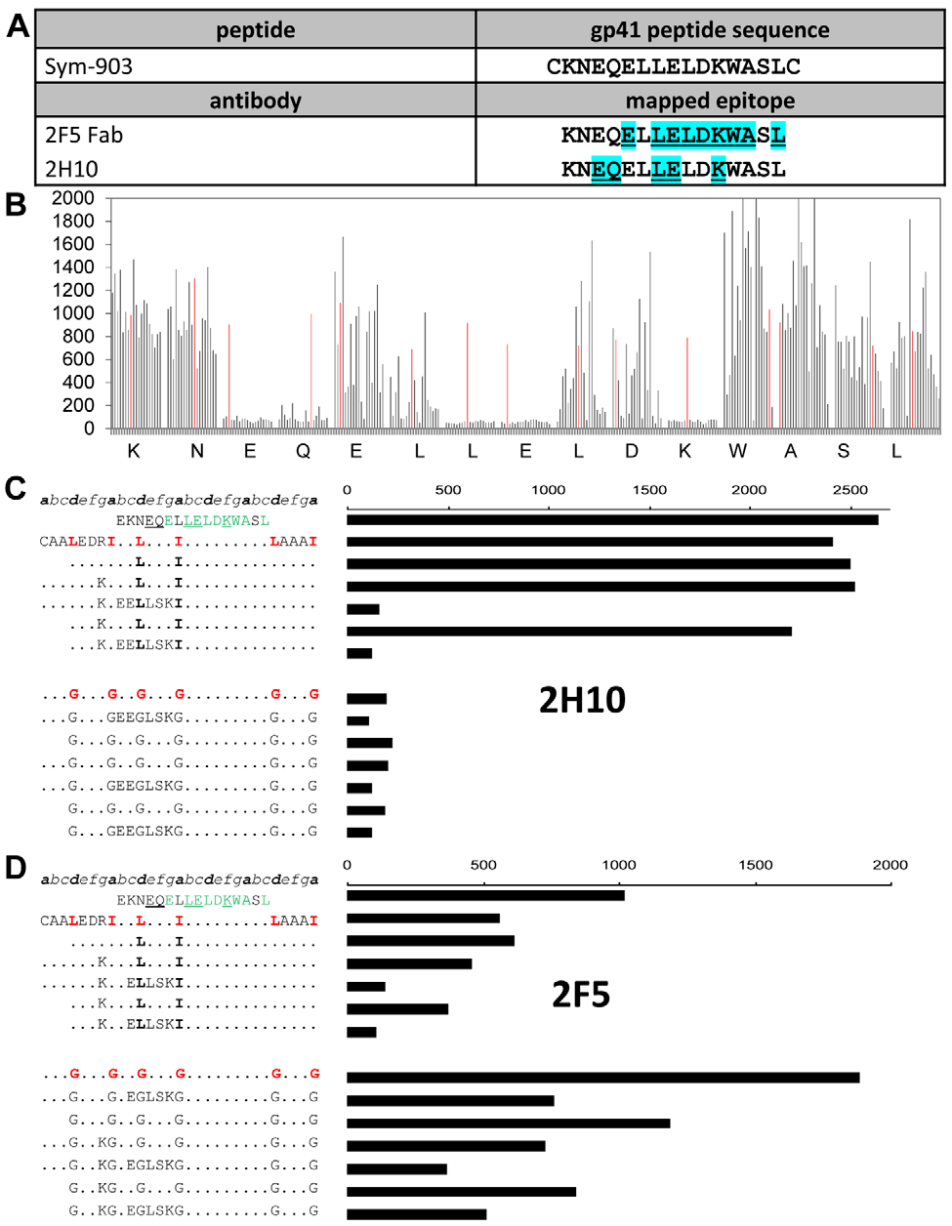
Epitope mapping of 2H10 and binding to helical peptides. **A**) The peptide Sym-903 was used for the replacement analysis with 2F5 and 2H10. Underlined cyan colored residues are recognized by the antibodies. **B**) Full substitution analysis of 2H10 epitope on gp41 MPER. The binding activity of VHH 2H10 at 2 ng/ml with a peptide is shown as a vertical line proportional to the Pepscan ELISA signal. Each group of 19 lines corresponds to the replacement set of all residues except cysteine for each amino acid position in the original 15-mer cyclic peptide: KNEQELLELDKWASL. Within each group of 19 lines the substitutions are in alphabetical order: based on the one-letter amino acid code (ADEFGHIKLMNPQRSTVWY) and the reactivity of the original nonapeptide is shown as a red line. The peptides contained an N- and C-terminal cysteine and were cyclised by addition of a T2-CLIPS [115]. These results show that EQ at position 3 and 4 and LE at position 7 and 8 and K at position 11, cannot be replaced by another amino acid without loss of binding and are thus essential for binding 2H10. **C**) Binding of VHH 2H10 (100 ng/ml) and **D**) Mab 2F5 (1 ng/ml) to gp41-MPER peptides grafted in GCN4-like helical templates that contain a stabilizing Ile/Leu heptad repeat (top panel) and binding to peptides in which the zipper motif is mutated to destabilizing glycine residues (lower panel). Peptide sequences are all based on hybrids of HIV-1 gp41 and GCN4. Alignment is shown of peptide sequence with heptad repeat positions (top line). Residues essential for 2H10 binding according to the substitution analysis are underlined and residues essential for 2F5 binding are in green. Mutations are shown with one-letter codes and dots indicated that the residue is not changed.

To determine the epitope of 2H10, its binding to a set of overlapping linear and cyclic peptides covering the gp41 MPER was measured. The best binding cyclic 15-mer peptide covered the gp41 region from amino acid 655 to 669 (_655_KNEQELLELDKWASL_669_). This peptide was used as a seed for a library in which the amino acids on each position of the peptide was substituted by all other 19 natural amino acids. Probing this library with 2H10 and 2F5 Fab allowed the mapping of the 2H10 epitope and identified five residues (E657, Q658, L661, E662 and K665) important for 2H10 binding. Notably, three out of the five are part of the 2F5 epitope (Fig. 3A and 3B).

Next, we tested whether 2H10 recognizes its epitope in a helical conformation. To achieve this, the peptide sequence (residues 655–669) was fused to a short coiled-coil that was shown to favor a helical conformation of the fused sequences [103]. The fusion construct was designed in such a way that the 2H10 residues required for interaction are exposed. Binding assays demonstrated that 2H10 binding was unaffected when the MPER peptide was fused to the coiled-coil template (Fig. 3C). In contrast, 2F5, which binds its epitope in a β-hairpin conformation [24] shows a dramatically reduced binding to the coiled-coil MPER construct (Fig. 3D). Replacement of heptad positions by Gly, which are helix breakers and thus do not support a helical MPER conformation, further leads to the loss of 2H10 interaction (Fig. 3C).

### 2H10 binding to Env and construction of bivalent 2H10

**Figure 4:**
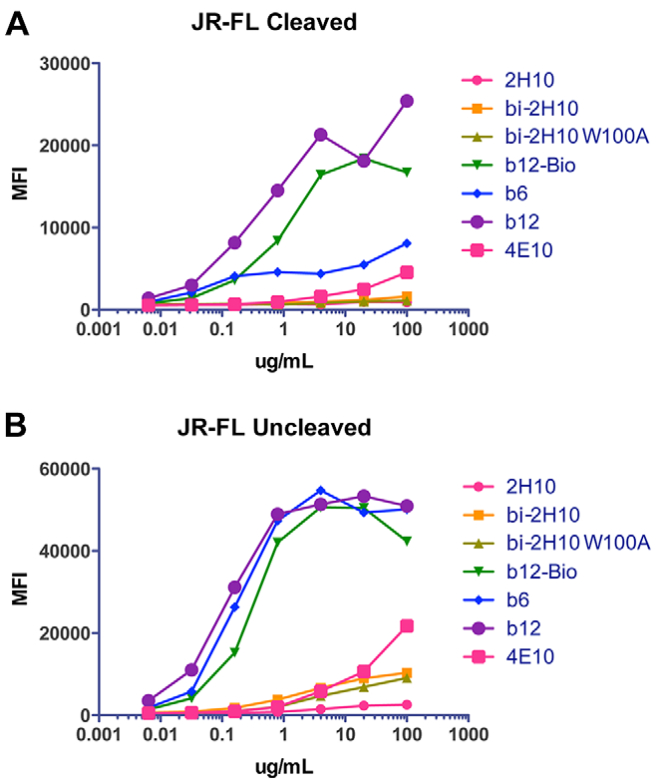
2H10, bi-2H10 and bi-2H10 W100A do not interact with membrane anchored Env. FACS analysis of HEK293T cells transfected with JR-FL Env in its cleaved (A) and un-cleaved form (B). The increase in mean fluorescence intensity (MFI) has been blotted as a function of antibody concentration. Control antibodies include b12, b6, b12-Bio (biotinylated version of B12) and 4E10. The lack of significant b6 interaction with the cells expressing cleaved Env indicates that most Env has been processed.

Because MPER antibodies primarily target the fusion intermediate state of gp41 [60], [104], [105], binding of 2H10 was tested to a soluble form of the fusion intermediate conformation of gp41, gp41_INT_ [60]. SPR measurements revealed a K_D_ of 29 nM for the 2H10 monohead (Fig. S1A). The avidity increased substantially to 2.5 nM, when two 2H10 VHH were linked with a 15-GS linker (bi-2H10) (Fig. S1B). Surprisingly, 2H10 and bi-2H10 interacted with similar affinities of 18 nM and 1.1 nM, respectively with gp41_523–883_, a potential late fusion intermediate conformation [29] (Figs. S1C and S1D). In addition binding to gp140 from clade 92UG037 was recorded. 2H10 interaction with gp140 was fit with a two state reaction model, because an experiment with different injection durations with 2H10 indicated that it binds in two states, a low affinity followed by a high affinity state (Fig. S1E), which may be due to heterogeneous conformational states of gp41 present in recombinant gp140 consistent with the presence of non-native Env on virions [106]. Again the affinity of 2H10 (K_D_ = 4 nM) is inferior to bi-2H10 binding (K_D_ = 0.15 nM), based on both the heterogeneous ligand fit and the two state reaction fit (Figs. S1F and S1G). In order to confirm the pepscan 2H10 binding to the linear gp41 peptide epitope, isothermal titration calorimetry was used to determine the affinity of 2H10 to linear and cyclic forms of peptide _655_KNEQELLELDKWASL_669_. This revealed affinities of 35 nM and 84 nM, respectively, indicating that cyclisation of the peptide epitope is unfavorable and reduces the binding affinity. However, the K_D_ of 35 nM is in good agreement with the SPR data and indicates that no other determinants of gp41 (present in gp41_int_) contribute to binding. We next tested whether 2H10 interacts with wild type native Env expressed on the plasma membrane, which revealed no interaction with either cleaved or uncleaved forms of JR-FL Env comparable to the lack of significant 4E10 interaction (Fig. 4). We conclude that 2H10 interacts with a linear sequence with a helical conformation present in two gp41 conformations tested.

### Bi-2H10 neutralizes HIV-1

The 2H10 VHH monohead did not neutralize any of the HIV-1 strains tested (Table 1). However, bivalent 2H10, which showed an increased affinity for gp41 as compared to the monohead, was able to neutralize HIV-1 isolates and pseudoviruses in a TZM-bl assay. No substantial difference was observed for bi-2H10 with a 15 or 17 amino acid linker. The activity of bi-2H10 with the 17 amino acid linker (bi-17GS) was mainly directed towards clade B, neutralizing 14 of 26 B isolates, one clade A (i.e. 92UG037) and none of the clade C isolates due to the sequence conservation of the epitope. Eight strains neutralized are Tier 1 and seven are Tier 2 viruses. Bi-2H10 also neutralized both the neutralization sensitive and resistant variants of SHIV SF162 consistent with SF162.LS neutralization (Table 1). The IC50 values were between equal to and 100-fold lower than those of mAb 2F5, which neutralized 18 out of 23 clade B viruses tested (Table 1). Bi-2H10, like 2H10, did not interact with cell surface expressed JR-FL Env (Fig. 4), although the virus was neutralized. We conclude that that bi-2H10 acts downstream of receptor-induced conformational changes in Env and the increased avidity of bi-2H10 renders the VHH neutralization active.

**Table 1:** 2H10, bivalent 2H10 and mutant bi-2H10 IC50 titers against HIV-1 in TZM-bl cells.

						IC50 (µg/ml)				
HIV-1/SHIV strain	Type	Clade	Tier	Accession code	Epitope sequence	2H10	bi-15GS	bi-17GS	W100A bi-15GS	2F5
HXB2	PV	B	1	K03455	**EQ**EL**LE**LD**K**					
BaL.26	PV	B	1	DQ318211	**EQ**EL**LE**LD**K**	•	6.9	11	•	10
SS1196.1	PV	B	1	AY835442	**EQ**EL**LE**LD**K**	•	13.5	13	•	15.5
NL4_3	PV	B	1	U26942	**EQ**EL**LE**LD**K**	•	4.4	0.2	•	0.087
IIIB	TCLA	B	1	EU541617	**EQ**EL**LE**LD**K**	•	9.0	15.7	•	0.4
AC10.0.29 (SVPB13)	PV	B	2	AY835446	**EQ**EL**L**ALD**K**	•		•		4.4
RHPA4259.7 (SVPB14)	PV	B	2	AY835447	**EQ**EL**L**ALD**K**	•		•		13.46
SF162.LS	PV	B	1a	EU123924	**EQ**EL**LE**LD**K**	•	49.02		•	1.77
MN-3	PV	B	1	AY669737	**EQ**EL**LE**LD**K**	•	0.78		4.80	<0.02
JR-FL.JB	PV	B	2	AY669728	**EQ**EL**LE**LD**K**	•	2.42		15.01	3.52
6535.3	PV	B	2	AY835438	**E**LEL**LE**LD**K**	•	•		•	5.86
WITO4160.33	PV	B	2	AY835451	**E**LEL**LE**LD**K**	•	•		•	1.05
TRJO4551.58	PV	B	3	AY835450	**E**LEL**L**KLDQ	•	•		•	•
REJO4541.67	PV	B	2	AY835449	**EQ**EL**LE**LD**K**	•	•		•	1.04
PVO.4	PV	B	3	AY835444	**EQ**DL**L**ALD**K**	•	•		•	•
TRO.11	PV	B	2	AY835445	**EQ**EL**LE**LDS	•	•		•	•
CAAN5342.A2	PV	B	2	AY835452	**E**KEL**LE**LD**K**	•	•		•	10.11
THRO4156.18	PV	B	2	AY835448	**E**KEL**LE**LD**K**	•	33.48		•	•
QH0692.42	PV	B	2	AY835439	**E**HEL**LE**LD**K**	•	14.35		•	1.01
SC422661.8	PV	B	2	AY835441	**EQ**EL**LE**LD**K**	•		10.8		
SHIV SF162P4	PV	B	1	M65024	**EQ**EL**LE**LD**K**	•		29.3		
SHIV SF162P3	PV	B	2	AY988107	**EQ**EL**LE**LD**K**	•		4.4		
WEAU-d15.410.787	PV	B(T/F)	2	EU289202	**EQ**EL**LE**LD**K**	•	4.76		17.21	0.59
1006-11.C3.1601	PV	B(T/F)	2	EU289183	**E**LEL**L**ALD**K**	•	•		•	1.99
1054-07.TC4.1499	PV	B(T/F)	2	EU289185	**E**KEL**LE**LD**K**	•	•		•	•
1056-10.TA11.1826	PV	B(T/F)	1B	EU289186	**EQ**EL**LE**LD**K**	•	28.9		•	0.26
1012_11_TC21_3257	PV	B(T/F)	1B	EU289184	**E**KEL**LE**LD**K**	•	•		•	0.91
92UG037.A9	MC	A	ND	AB253429	**E**KDL**LE**LD**K**	•	2.4	28	•	
92UG0378	PV	A	2	AB253429	**E**KDL**LE**LD**K**	•	4.66		18.69	0.60
Q23.17	PV	A	ND	AF004885	**E**KEL**LE**LD**K**	•	•		•	4.97
Q769.d22	PV	A	2	AF407158	**EQ**DL**L**ALD**K**	•	•		•	4.92
0330.v4.c3	PV	A	ND	HM215257	**EQ**DL**L**ALD**K**	•	•		•	14.10
ZM249M.PL1	PV	C	2	FJ496214	**E**KDL**LE**LDS	•	•		•	•
ZM135M.PL10a	PV	C	2	AY424079	**E**KDL**L**ALDS	•	•		•	•
ZM53M.PB12	PV	C	2	AY423984	**E**KDL**L**ALDS	•	•		•	•
93MW965.26	PV	C	1	U08455	**E**KDL**L**ALDS	•		•		
96ZM651.02	PV	C	2	AF286224	**E**KDL**L**ALDS	•		•		•
ZM 109F.PB4	PV	C	2	AY424138	**E**KEL**L**ALN**K**	•		•		

Neutralization activities of 2H10, bivalent 2H10, W100A mutant bi-2H10 and as a control 2F5 were determined against the indicated viruses. The type, clade, tier and the accession code of the envelope protein sequence are given for each virus strain. The wild-type bi-2H10s are named bi-15GS and bi-17GS and have a 15 and 17 residue linker respectively. The W100A mutant bi-2H10 is named W100A bi-15GS and has a 15 residue linker. The column labeled Tier indicates whether the virus is classified for 1,2, or 3 assessment of neutralizing antibodies [116]. The column labeled Epitope sequence, shows the sequence that aligns with the part of the HBX2 sequence to which 2H10 is directed. The underlined, boldfaced residues are essential for the binding of 2H10 and are equal to the residues at the equivalent positions in HXB2, which was used for immunization. Residues in grey are at the position of an essential residue, but deviate from the HBX2 equivalent. • indicates IC50>50 µg/ml; the empty cells represent non-performed measurements.

### 2H10 exposes W100 at the tip of CDR3 which is required for neutralization

**Figure 5:**
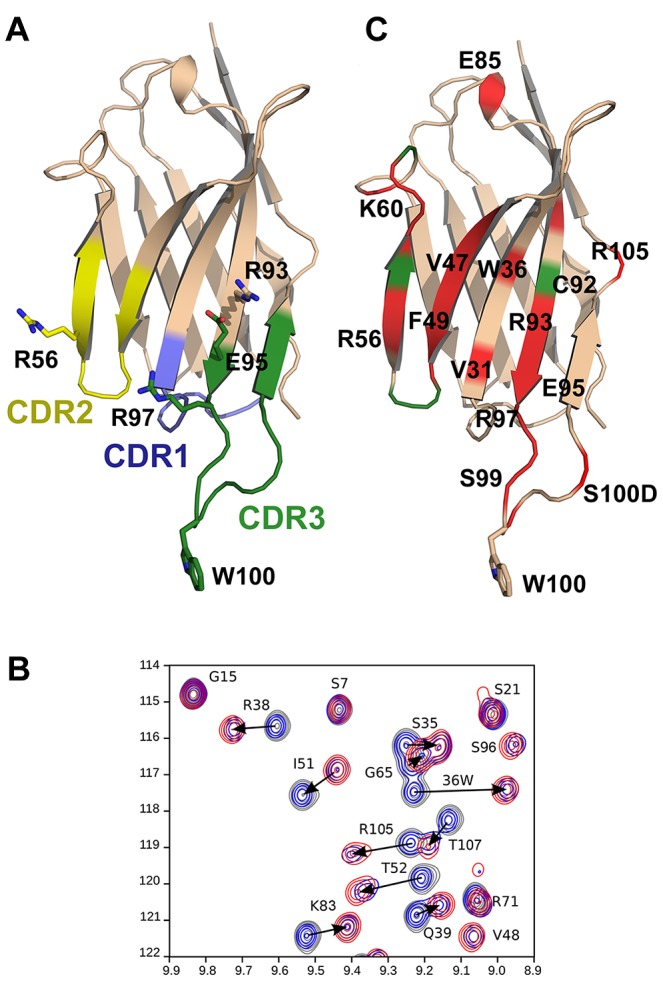
Crystal structure of 2H10 and NMR analysis of 2H10 interaction with the gp41 peptide. **A**) Ribbon representation of 2H10. CDR 1, 2 and 3 are colored blue, yellow and green, respectively. Residues implicated in gp41 interaction by mutagenesis are shown as sticks. W100 at the tip of CDR3 is required for neutralization. **B**) Selected region of the ^15^N HSQC spectra (see Fig. S3) recorded on a ^13^C,^15^N labeled 2H10 sample. Increasing concentrations of the gp41 peptide were titrated into the 2H10 solution and induced specific chemical shifts; spectra were recorded with a protein∶peptide ratio of 1∶0, in grey, 1∶0.5 in blue and 1∶1 in red.**C**) Chemical shift perturbations (CSPs) were mapped onto the 2H10 structure. Residues showing ^15^N,^1^H chemical shift perturbations greater than 0.15 ppm are shown in red and residues with an amide resonance disappearing in the free or the bound form are colored in green.

**Figure 6:**
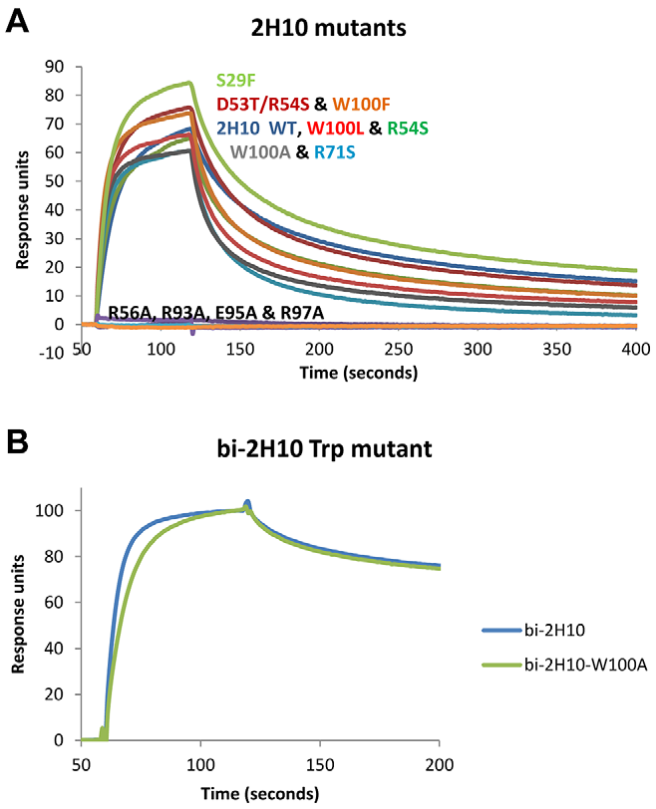
Surface plasmon resonance affinity measurements reveal essential and non-essential 2H10 residues for gp41 interaction. **A**) 2H10 single domain mutants. The curves are labeled with the mutant codes in colors corresponding with the colors of the curves. The mutants R56A, R96A, E98A and R100A do not bind at all to gp140-92UG037. Fitting the curves with the two-state reaction algorithm yielded K_D_s of 8.0 nM for 2H10 WT and 10.1 nM for 2H10-W100A. The other fits, except for those of the non-binding mutants, yielded K_D_s between 5.5 nM for 2H10-S29F and 18.7 nM for 2H10-R71S **B**) bi-2H10-W100A mutant and wild type bi-2H10 on immobilized gp140-92UG037. Because the maximum responses units are slightly different for the two bi-2H10 molecules, the curves were normalized to the maximum response, to be able to compare the dissociation rates well.

To study the interaction of 2H10 with its epitope, several attempts were made to obtain crystals of 2H10 in complex with various MPER peptides based on the Pepscan analyses. However, in each attempt, diffraction quality crystals were obtained that contained the VHH, but not the peptide. The best 2H10-containing crystals diffracted to 1.3 Å resolution and the structure was solved by molecular replacement (Table 2). The structure resembles the framework of known VHH structures [76], with the major exception that most other VHH structures have CDR3 loops that fold back on the framework of the VHH, whereas the CDR3 of 2H10 protrudes from the framework of 2H10. However, it should be noted that its orientation may have been influenced by crystal contacts. Its most notable feature is the exposure of W100 at the tip of CDR3 (Fig. 5A). CDR3 W100 resembles the presence of hydrophobic residues within the CDR3 heavy chains of 2F5 and 4E10 [24], [25], which are not essential for epitope interaction although changes in affinity have been reported depending on the nature of the antigen for some mutants. In contrast, mutations of hydrophobic CDR3 residues affect the neutralization potency of these antibodies dramatically [48]–[50]. Similarly, several 2H10 W100 mutants (i.e. W100A, W100F and W100L) interacted with gp41_INT_ and gp140 with affinities comparable to wild type 2H10 (Fig. 6A and Fig. S1H), confirming that W100 is non-essential for epitope interaction. To study whether W100 is required for neutralization, the W100A mutation was introduced into bi-2H10 (bi-2H10-W100A). SPR confirmed that bi-2H10-W100A binds to gp140 comparable to wild type bi-2H10 revealing the same dissociation rate together with a slightly slower on-rate (Fig. 6B). Although not essential for gp41 interaction, the exchange of W100 to alanine completely abrogated neutralization of most strains. Four other strains tested showed a reduced potency as evident from the 4-and 6-fold increased IC50 values for these viruses (Table 1). In addition, bi-2H10 W100A did not interact with surface-expressed Env as expected (Fig. 4). We thus conclude that CDR3 W100 is dispensable for epitope interaction but is essential for neutralization similar to the role of hydrophobic CDR3 heavy chain residues present in mAbs 2F5 and 4E10 [48]–[50]. In order to map the gp41 binding site on 2H10 the following mutants (S29F, D53T/R54S, R54S, R56A, R71S, R93A, E95A, R97A) were designed based on the structure (Fig. 5A) and the VHH germline sequence of CDR1 and CDR2 (Fig. 2). Assessment of binding by SPR revealed that the single mutants R56A, R93A, E95A, and R97A lost binding to gp140-92UG037 (Fig. 6A). All other mutants (S29F, D53T/R54S, R54S, R71S) still bound gp140, although the affinities for some seemed to be marginally lower than for wild-type 2H10. This thus implicates the charged residues R56 in CDR2 and R93, E95 and R97 of CDR3 in gp41 recognition.

**Table 2:** Data collection and refinement statistics.

Space group		I23
Cell dimensions	*a*, *b*, *c* (Å)	89.95, 89.95, 89.95
Wavelength (Å)		0.9395
Resolution (Å)		25.00–1.3 (1.3–1.37)
Measured reflections		3427903
Unique reflections		38741
*R*merge		0.069 (0.295)
*I*/σ*I*		6.1 (2.2)
Completeness (%)		99.0 (100.0)
Redundancy		13.9 (14.2)
Wilson B factor (Å^2^)		12.2
*R*cryst (N° reflections)		0.14 (29381)
*R*free (N° reflections)		0.17 (1509)
No. atoms	Protein	4960
	Water	172
*B*-factors	Protein	32.32
	Water	33.89
R.m.s deviations	Bond lengths (Å)	0.005
	Bond angles (°)	1.09

Numbers in parentheses refer to the highest resolution shell. R_merge_ = Σ|I _ 〈I〉|/〈ΣI〉, where I = observed intensity. R_cryst_ = Σ|Fo _ Fc|/Σ|Fo|, where |Fo| = observed structure factor. Amplitude and |Fc| = calculated structure factor amplitude. R_free_ is R_cryst_ for 3% of reflections excluded from the refinement.

Because it was reported that mAbs 2F5 and 4E10 have lipid binding activity, we tested whether 2H10 also binds liposomes or lipids. Sucrose gradient flotation analyses showed that 2H10 does not float with liposomes having the lipid composition of native HIV-1 virions. 2H10 is only found in the bottom fractions of the gradient while a substantial amount of 4E10 floats to the upper fraction of the gradient in this assay. However, 2F5 did not reveal any liposome binding either (Fig. S2A). This is consistent with ELISA data which demonstrated binding of mAb 4E10 to cardiolipin and phosphatidylserine, while 2H10, bi-2H10 and 2F5 revealed no binding activity (Fig. S2B). We conclude that 2H10 exerts no measurable lipid binding activity.

### 2H10-gp41 complex formation: NMR characterization of 2H10 in the free and peptide-bound form

^15^N HSQC spectra of 2H10 were recorded in the absence or presence of an excess of the gp41 peptide and revealed a sufficient spectral resolution to allow the 1H,15N, and 13C backbone assignment of the two forms using heteronuclear experiments (Fig. S3). Chemical shift index (CSI) calculated from the CB, CA, N and CO chemical shifts reveal similar secondary structures for the free and the bound forms (Fig. S4). The specific shift of individual residues was observed using ^15^N HSQC spectra recorded at different ratios of gp41 peptide to 2H10 and depend on the peptide concentration (Fig. 5B). These data reveal a slow exchange process between the bound and the free form in agreement with the affinity constant measured for this complex. The complete list of chemical shift differences (CSD) induced in 2H10 upon peptide binding is listed in Figure S5 (Fig. S5), which confirmed further that peptide binding, did not induce conformational changes in the tertiary structure of 2H10. Notably most significant CSDs map to one surface of the 2H10 structure providing a first clue on the gp41 ligand position (Fig. 5C).

### Modeling of the 2H10 gp41 peptide complex

**Figure 7:**
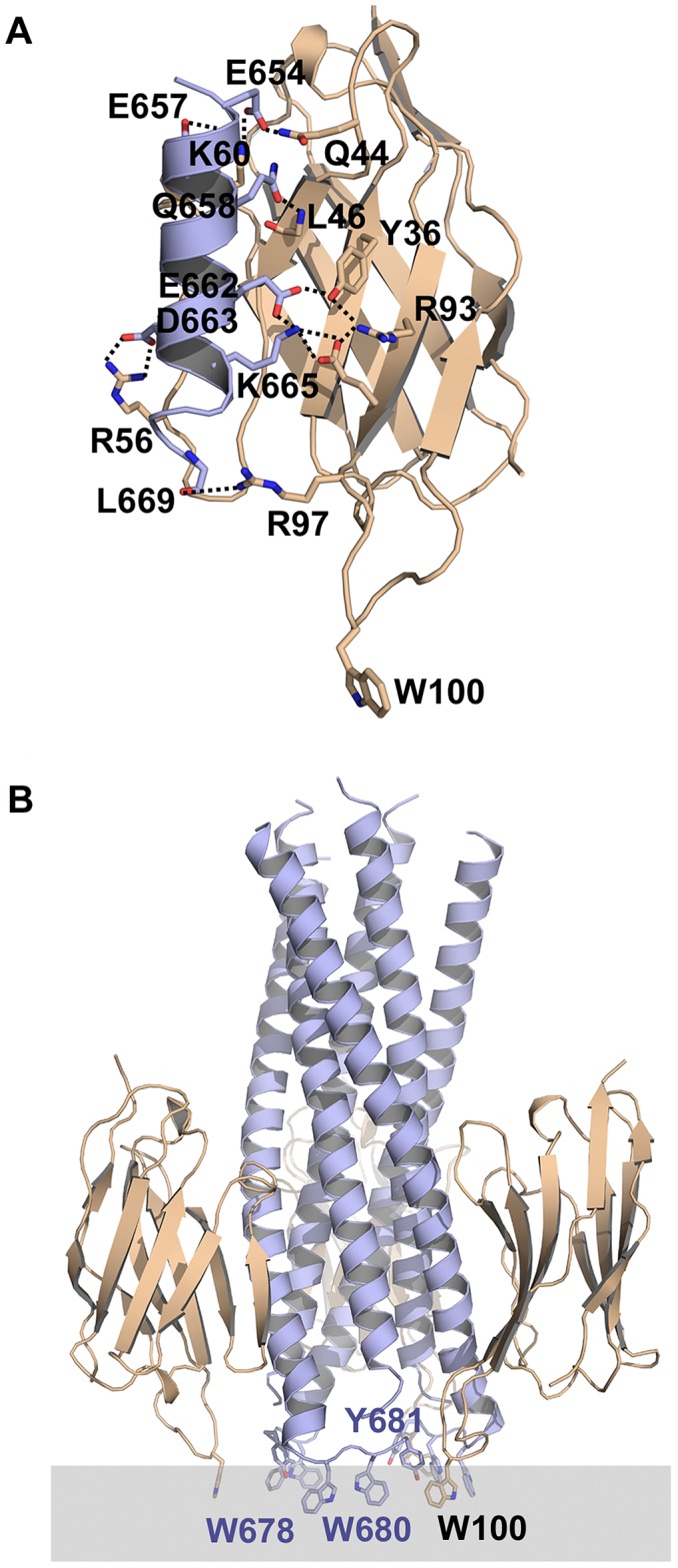
Molecular modeling of the 2H10-gp41 peptide interaction suggests that 2H10 W100 is oriented towards the membrane. **A**) Molecular model of the gp41 peptide interaction with 2H10 produced by HADDOCK. Salt bridges and hydrogen bonds present in the top model and found in most of the top10 models are shown as dashed lines. **B**) Superpositioning of the Cα atoms of the gp41 peptide derived from the HADDOCK 2H10-peptide model onto the structure of a late fusion intermediate of gp41 [29] shows that the CDR3 W100 is oriented towards the membrane and found in the same plane as gp41 residues W678, W680 and Y681. Note that there are no clashes between the 2H10 VHH and gp41.

Modeling of the 2H10 interaction with a helical gp41 peptide conformation with HADDOCK [97] readily produced a top cluster with an average cluster score significantly lower than any other solution (−96.3±0.5 a.u.). This cluster corresponded also to the most populated one with 173 members out of 200 models generated. Its electrostatic energy is −437.0±31.1 kcal/mol and its Van der Waals energy −36.9±6.9 kcal/mol. The model reveals that the short gp41 helix binds in a parallel fashion to the 2H10 beta-sheet involving a network of salt bridges (E654-K60; K665-E95; D664-R56) and hydrogen bonds (E654-Q44; E657-S62 amide; Q658-amide-L47; E662-Y37; R96-L669 carbonyl). In addition, R93 stabilizes the orientation of Y36 and E95, which contact E662 and K665, respectively (Fig. 7A). The network of interactions is consistent with the chemical shift perturbation (Fig. 5B) and mutagenesis data, which implicate R56, R93, E95 and R97 in the interaction. CDR3 S99 and S100D show additional chemical shift differences upon peptide binding. This may indicate that the CDR3 may be flexible enough to contact the peptide or alternatively, that peptide binding induces major changes within the CDR3 conformation or orientation. Among the interacting residues are five of the six framework residues that deviate from the germline (S35, F50, K60, V61 and R93) by at least two nucleotide changes in their codons (Fig. 2B). The sixth residue (L91) is very close to the interaction site. Their occurrence in VHH is rather rare (F50,1.5% of VHH sequences, K60, 0.8%, V61, 0.7% and R93, 0.52% of VHH sequences (https://fungen.wur.nl/). The model also corroborated that CDR3 W100 does not interact with gp41. Subsequent superpositioning of the Cα atoms of the VHH-peptide model (Fig. 7A) and the trimeric structure of the late fusion intermediate [29] yielded a trimer model without any clashes (Fig. 7B). Importantly, it revealed that the side chain of W100 is positioned in such a way that it could dip into the membrane bilayer in the same plane as gp41 MPER residues W678, W680 and Y681 (Fig. 7B). The latter have been proposed to insert into the membrane during the conformational changes of gp41 leading to membrane fusion [29]. We conclude that 2H10 binds a helical epitope of gp41, which allows positioning of the CDR3 W100 towards the membrane. Due to the small size of the VHH, 2H10 may stay associated with gp41 until late stages of membrane fusion.

## Discussion

The HIV-1 envelope fusion subunit gp41 contains highly conserved epitopes within its MPER that are recognized by the potent and broadly neutralizing antibodies 2F5 and 4E10. Although these bnAbs recognize linear epitopes, immunization with gp41 peptides or proteins has not yet produced such antibodies [107]. In this study, we isolated and characterized a VHH from a llama immunized with gp41 proteoliposomes. Similar gp41 proteoliposomes, with a different liposome formulation and gp41 construct, used in a previous immunization study of mice using a different immunization strategy, yielded sera with no significant neutralization [37]. The VHH 2H10 recognizes an epitope (**EQ**EL**LE**LD**K**) that partially overlaps with the 2F5 epitope [20], [24] and binds to its epitope present in various Env conformations with low nanomolar affinity.

One hypothesis is that neutralizing antibodies directed against MPER target epitopes that are only transiently exposed during the entry process [60], [104]. Consequently their binding to the native Env structure may be weak, as demonstrated for mAb 2F5, which interacts only with few Env gp140 oligomers [60] and the absence of significant binding of MPER antibodies to membrane-anchored Env with the exception of 10E8 [9]. Because neither monomeric 2H10 nor bi-2H10 interacted with cleaved or uncleaved JR-FL Env, we hypothesize that their target is an Env conformation induced by receptor binding. In contrast 2H10 binds to recombinant Env gp140 derived from strain 92UG037. Binding of 2H10 is best described by a two-state reaction of a low affinity contact followed by a high affinity interaction. Thus 2H10 may induce its epitope in the soluble version of gp140, which may be facilitated by the fact that the C-terminal end of this recombinant gp140 protein is not constrained by the transmembrane region. Due to the potentially transient nature of the 2H10-epitope exposure, we tested binding to a soluble form of the fusion intermediate conformation of gp41, gp41_INT_ [108]. SPR measured a 30 nM affinity for 2H10 binding to gp41_INT_, which is approximately 30 times lower than the 2F5 Fab affinity for the fusion intermediate conformation of gp41 from strain 92UG037, 92UG-gp41-inter-Fd [60]. Bi-2H10 showed a more than tenfold increase in binding to gp41_INT_, but still a lower affinity when compared to the K_D_s of Fab or scFv interactions of 2F5 and 4E10 with 92UG-gp41-inter-Fd [60]. It is thus possible that the lower affinity of monovalent 2H10 for gp41_INT_ may affect its neutralization capacity of any of the tested HIV-1/SHIV strains. However, increasing the affinity by an increased avidity as demonstrated for bi-2H10 led to the neutralization of selected strains including some Tier 2 viruses with IC50 values ranging from 0.2 to 49 µg/ml, when using the stringent TZM-bl cell assay. Bi-2H10 may achieve HIV-1 entry inhibition by binding two gp41 molecules from the same trimer or cross link two gp41 trimers.

The crystal structure of the 2H10 VHH highlighted the longer than usual CDR3 that displays a solvent exposed tryptophan (W100) at its tip. Mutagenesis showed that W100 is not required for interaction with gp41_INT_ or gp140. However, bi-2H10 with a W100A mutation either no longer neutralized or showed a largely reduced neutralization potency (increases of IC50 values ∼6- and 4-fold) against Tier 1 and Tier 2 strains despite its wild-type-like interaction with gp41_INT_. The presence of W100 in the CDR3 of 2H10 is reminiscent of hydrophobic determinants within the CDR3 H3 regions of mAbs 2F5 and 4E10, which are not required for gp41 interaction, but are important for neutralization activity [48]–[50]. In case of mAb 2F5, single Ser substitutions resulted in a 15- to 500-fold reduction in neutralization potency, while double mutations to Ser completely abrogated 2F5-mediated neutralization [50]. Even though most HIV-1 strains were no longer neutralized by the bi-2H10 W100A mutant, it is conceivable that a W100 to Ser mutation may have been even more efficient in affecting neutralization due to the polar propensity of Ser. Similarly, single mutations in the CDR3 of mAb 4E10 reduced its neutralization potency [48] and it was noted that Asp substitutions exerted a greater effect on neutralization potency than Ala substitutions [49]. The effect of the bi-2H10 W100A mutant also suggests that neutralization by bi-2H10 is not solely due to heteroligation of two antigen binding domains via an increased avidity [59], [109]. We therefore propose that CDR3 W100 plays a crucial role in neutralization by bi-2H10, which resembles the role of the hydrophobic CDR3 of mAbs 2F5 and 4E10 in neutralization.

Numerous studies suggested that the hydrophobic CDR3 residues are implicated in lipid/membrane interaction thereby facilitating antigen binding [46], [110], [111]. One possibility is that MPER itself becomes membrane-embedded during the fusion reaction and MPER-specific antibodies need to recognize their epitope within the membrane context [52], [112]. We therefore presented MPER in a lipid bilayer context to allow selection of antibodies that can recognize lipid and protein determinants. However, despite the presence of W100 in CDR3, 2H10 as well as the control mAb 2F5 did not show any detectable lipid binding specificity *in vitro*.

All HIV-1 strains that were neutralized by bi-2H10 have the epitope sequence determined by pepscan analyses except for 92UG037.A9 which has Q658 replaced by a lysine. However, SPR measurements of 2H10 to gp140 env derived from 92UG037.A9 corroborate 2H10 binding. The discrepancy may be explained by the use of cyclic peptides in the pepscan analysis, which showed a lower affinity in the ITC experiment. Therefore a Q658K exchange may have had a larger effect on binding within the cyclic version than within natural contexts of the epitope. Sequence analysis of 2300 Env sequences revealed that ∼28% have the epitope 657-**E(Q/K/H)**-XX-**LE**-XX-**K**-665. Considering only B clades, the number of isolates covered would rise to ∼61%. In comparison, 2F5 and 4E10 have been shown to neutralize 68% and 97% of all clade B strains tested and 60% and 98% of all clades, respectively [1].

The combined structural approach using peptide modeling, NMR, docking and mutagenesis showed that 2H10 interacts with a helical linear gp41 epitope. VHH maturation played an important role in optimizing antigen binding, because somatic mutations involve both CDR and framework residues at the interaction site. High framework mutation rate has been observed in broadly neutralizing human antibodies as well [5]–[7], [113]. The helical epitope conformation is present in the late fusion intermediate conformation of gp41, gp41_528–683_ [29] that interacts with 2H10. This is unexpected, because 4E10 whose epitope is exposed and accessible in gp41_528–683_ [29] does not interact with this conformation due to molecular clashes of the light chain with gp41 heptad repeat region 1 [29]. The fact that the small size of 2H10 allows binding to gp41_528–683_ may constitute a disadvantage for using small llama VHH over complete antibodies; the VHH may thus less efficiently prevent refolding of gp41 into the six helical bundle post fusion conformation. Nevertheless, an interesting feature surfaced from the 2H10-gp41_528–683_ complex model. The model poses 2H10 CDR3 W100 in the same plane as gp41 MPER residues W678, W680 and Y681, which may insert into the membrane during the fusion process [29]. This indirectly corroborates the hypothesis that the necessity of W100 for HIV-1 neutralization is in agreement with the suggestion that part of MPER may be membrane embedded. Because MPER, as observed in the late fusion-intermediate gp41_528–683_ structure, may be already membrane-embedded at an earlier stage, anti-MPER antibodies such as 2H10 may act much earlier to block the fusion process. Alternatively, bi-2H10 binding may prevent membrane extraction of MPER, which is likely required to finalize membrane fusion [29].

The epitope of 2H10 overlaps with the helical epitope of mAb 13H11, which is, however, non-neutralizing due to the absence of a long hydrophobic heavy chain CDR3 [114]. The helical epitope of 2H10 is part of the helical epitope recognized by the broadly neutralizing anti-MPER mAb 10E8, isolated from a patient [9]. In this MPER conformation, which may represent the structure of MPER within native Env trimers, residues 656 to 683 form two short L-shaped helices of which the C-terminal helix provides most antibody binding contacts. Most notably, mAb 10E8 employs hydrophobic CDR3 residues Phe 100A and W100B for interaction with its epitope. Because mAb 10E8 displays no autoreactivity it presents a new class of potent anti-MPER antibodies [9].

The VHH 2H10, which was elicited by gp41 proteoliposomes as antigen stresses the importance of a membrane component in generating anti-MPER neutralizing antibodies. Although there have been many attempts to generate neutralizing MPER-specific anti gp41 antibodies in animals, no protein-based immunization scheme has produced sera or antibodies that neutralized HIV-1 in the stringent TZM-bl assay [16], [17], [107]. In this study, we have demonstrated that it is possible to elicit anti-MPER antibodies that neutralize HIV-1 by employing proteoliposomes containing the native Env transmembrane region that may orient MPER optimally with respect to the lipid bilayer, which in turn is important for antibody neutralization [55]. Although bi-2H10 lacks the potency and breadth of 2F5, 4E10 or 10E8, optimization of the immunization protocol such as longer immunization schemes which may produce more extensive somatic mutations could yield antibodies with higher breadth and potency.

## Supporting Information

Figure S1 **SPR with 2H10 and bi-2H10.** The following ligands were immobilized on a were immobilized on a CM5 chip: gp41INT A), B) and H), gp41528-683 C) and D), and gp140-92UG037 E), F) and G). Injections were performed with a range of 1 to 200 nM for 150 seconds each on both gp41 constructs, while for the immobilized gp140-92UG037, 6.25, 25, 100 and 400 nM for 60 seconds was used. The fitted curves are represented by black lines and the raw data are shown in color. The data were measured in triplicates and the curves superpose very well. A) Response for 2H10on gp41INT, fitted with separate association and dissociation (shown association fit). B) Response for bi-2H10 on gp41INT. The curves were fitted using 1∶1 Langmuir model. C), D). Response for 2H10 and bi-2H10 on gp41528-683 fitted using 1∶1 Langmuir model. E) Proof of two-state reaction of 2H10 binding to gp140 on gp140-92UG037. Injections were performed with 100 nM 2H10 with different injection durations. The curves were normalized to the maximum of obtained response units. The observation that the dissociation is dependent on injection duration indicates a two-state reaction binding. F) Response for 2H10 on gp140-92UG037. The curves were fitted with the two-state reaction algorithm. G) Response for bi-2H10 on gp140-92UG037. The curves were fitted with the heterogeneous ligand algorithm. H) Response for mutant 2H10-W100A on gp41INT, fitted with separate association and dissociation (shown association fit).
(TIF)

Figure S2 **Lipid binding test of 2H10, 4E10 and 2F5.** **A**) Sucrose gradient centrifugation analyses of 2H10, 4E10 and 2F5. The top and bottom of the gradient are indicated; hc, heavy chain; lc, light chain. **B**) ELISA with cardiolipin and phosphatidylserine. Binding of 4E10, 2F5, 2H10 and bi-2GH10 are indicated. Left panel binding to cardiolipin; right panel, binding to posphatidylserine.
(TIF)

Figure S3 **15N HSQC of 2H10 without and with a 1.5-fold excess of peptide.** The spectra were acquired at 600 MHz, T = 37 C. Protein concentration was 0.95 mM, in in 25 mM HEPES buffer and 75 mM NaCl (pH 6.7).
(TIF)

Figure S4 **Secondary structure propensity of the free and bound form of 2H10 determined from CB, CA, CO, N chemical shift analysis using the program TALOS+.** The chemical shift index (CSI) value of 1 corresponds to 100% helical conformation propensity and a value of −1 corresponds to a 100% β-strand conformation propensity.
(TIF)

Figure S5 **Histograms showing the value of the chemical shift difference (CSD) in 2H10 induced upon binding with the gp41 peptide (1.5 fold excess).** Residue numbers are indicated on the X axis (residues for which CSD is missing are prolines. The green and pale green bars with an arbitrary height represent residues whose NH correlation is not present in the 15N-HSQC spectrum for the free and bound form of 2H10, respectively (probably because of conformation exchange broadening). The CSI analysis of the free and bound form of 2H10 show that upon addition of an excess of ligand, the secondary structure of the protein does not change.
(TIF)
