# Supplementary figures and images for: A gp41 MPER-specific Llama VHH Requires a Hydrophobic CDR3 for Neutralization but not for Antigen Recognition
Source: PLoS Pathog. 2013 Mar 7;9(3):e1003202. doi: 10.1371/journal.ppat.1003202 (PMC3591319; doi:10.1371/journal.ppat.1003202)

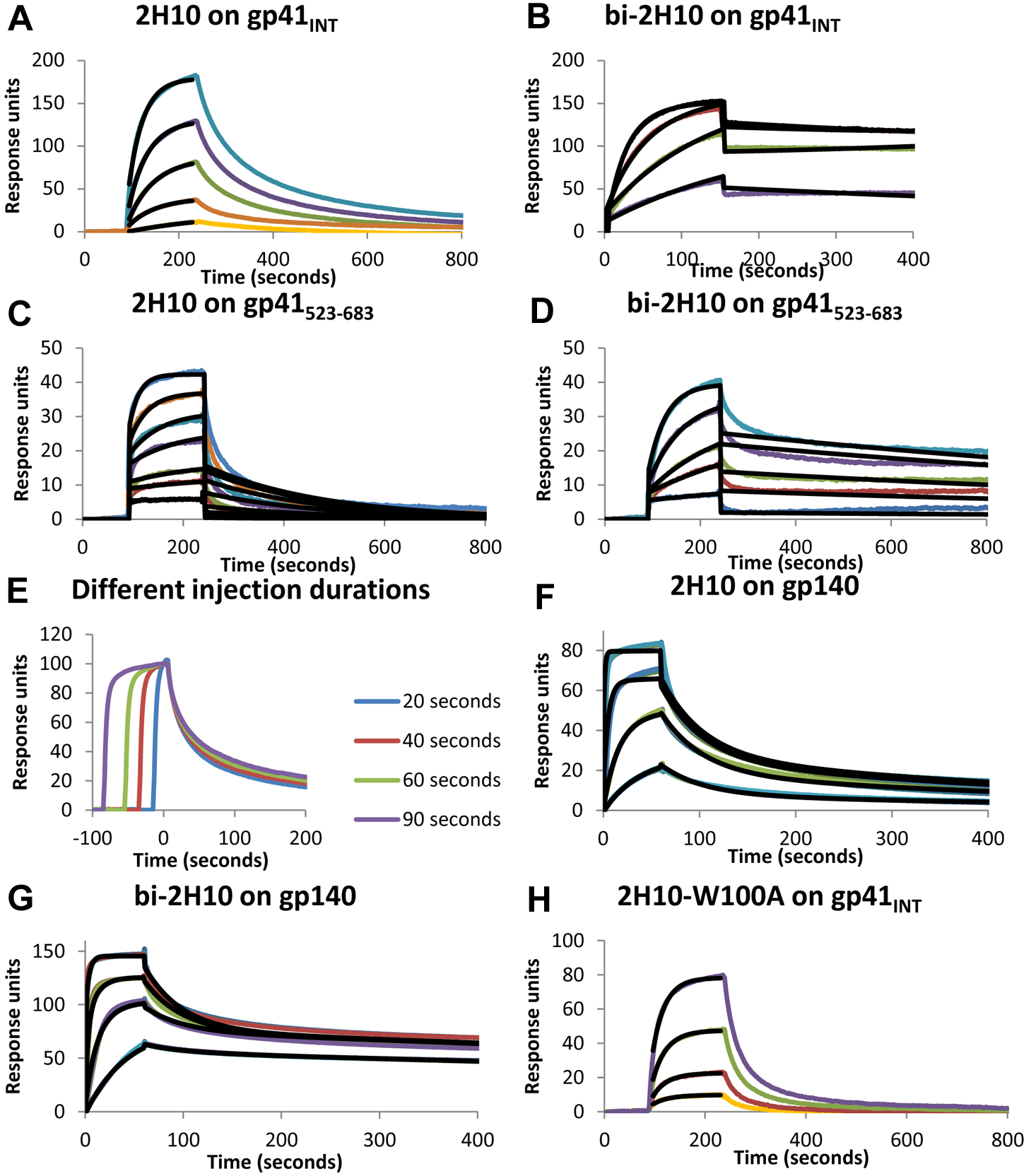

Supplement: Figure S1 — SPR with 2H10 and bi-2H10. The following ligands were immobilized on a were immobilized on a CM5 chip: gp41INT A), B) and H), gp41528-683 C) and D), and gp140-92UG037 E), F) and G). Injections were performed with a range of 1 to 200 nM for 150 seconds each on both gp41 constructs, while for the immobilized gp140-92UG037, 6.25, 25, 100 and 400 nM for 60 seconds was used. The fitted curves are represented by black lines and the raw data are shown in color. The data were measured in triplicates and the curves superpose very well. A) Response for 2H10on gp41INT, fitted with separate association and dissociation (shown association fit). B) Response for bi-2H10 on gp41INT. The curves were fitted using 1∶1 Langmuir model. C), D). Response for 2H10 and bi-2H10 on gp41528-683 fitted using 1∶1 Langmuir model. E) Proof of two-state reaction of 2H10 binding to gp140 on gp140-92UG037. Injections were performed with 100 nM 2H10 with different injection durations. The curves were normalized to the maximum of obtained response units. The observation that the dissociation is dependent on injection duration indicates a two-state reaction binding. F) Response for 2H10 on gp140-92UG037. The curves were fitted with the two-state reaction algorithm. G) Response for bi-2H10 on gp140-92UG037. The curves were fitted with the heterogeneous ligand algorithm. H) Response for mutant 2H10-W100A on gp41INT, fitted with separate association and dissociation (shown association fit). (TIF) [file ppat.1003202.s001.tif]

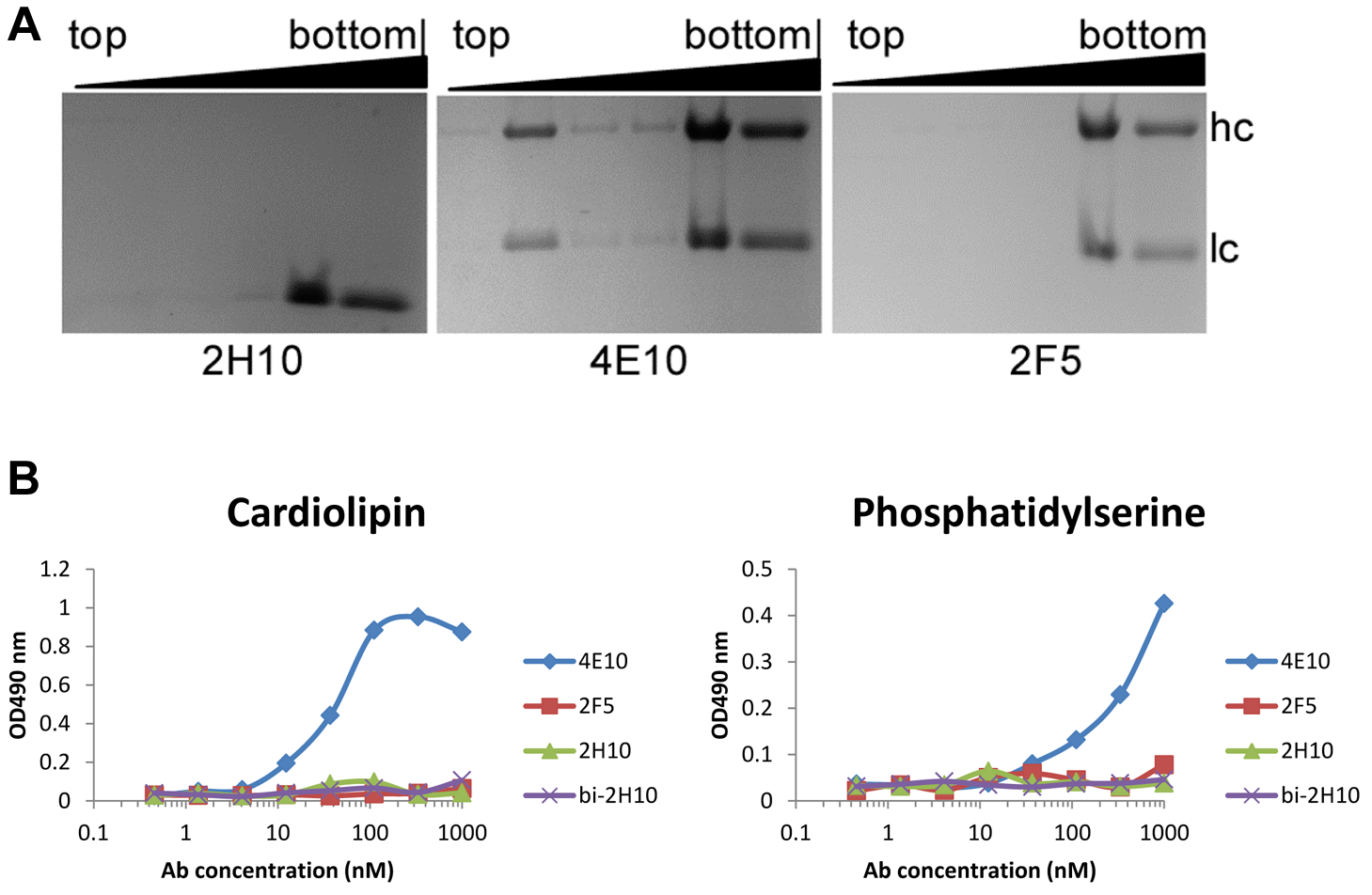

Supplement: Figure S2 — Lipid binding test of 2H10, 4E10 and 2F5. A) Sucrose gradient centrifugation analyses of 2H10, 4E10 and 2F5. The top and bottom of the gradient are indicated; hc, heavy chain; lc, light chain. B) ELISA with cardiolipin and phosphatidylserine. Binding of 4E10, 2F5, 2H10 and bi-2GH10 are indicated. Left panel binding to cardiolipin; right panel, binding to posphatidylserine. (TIF) [file ppat.1003202.s002.tif]

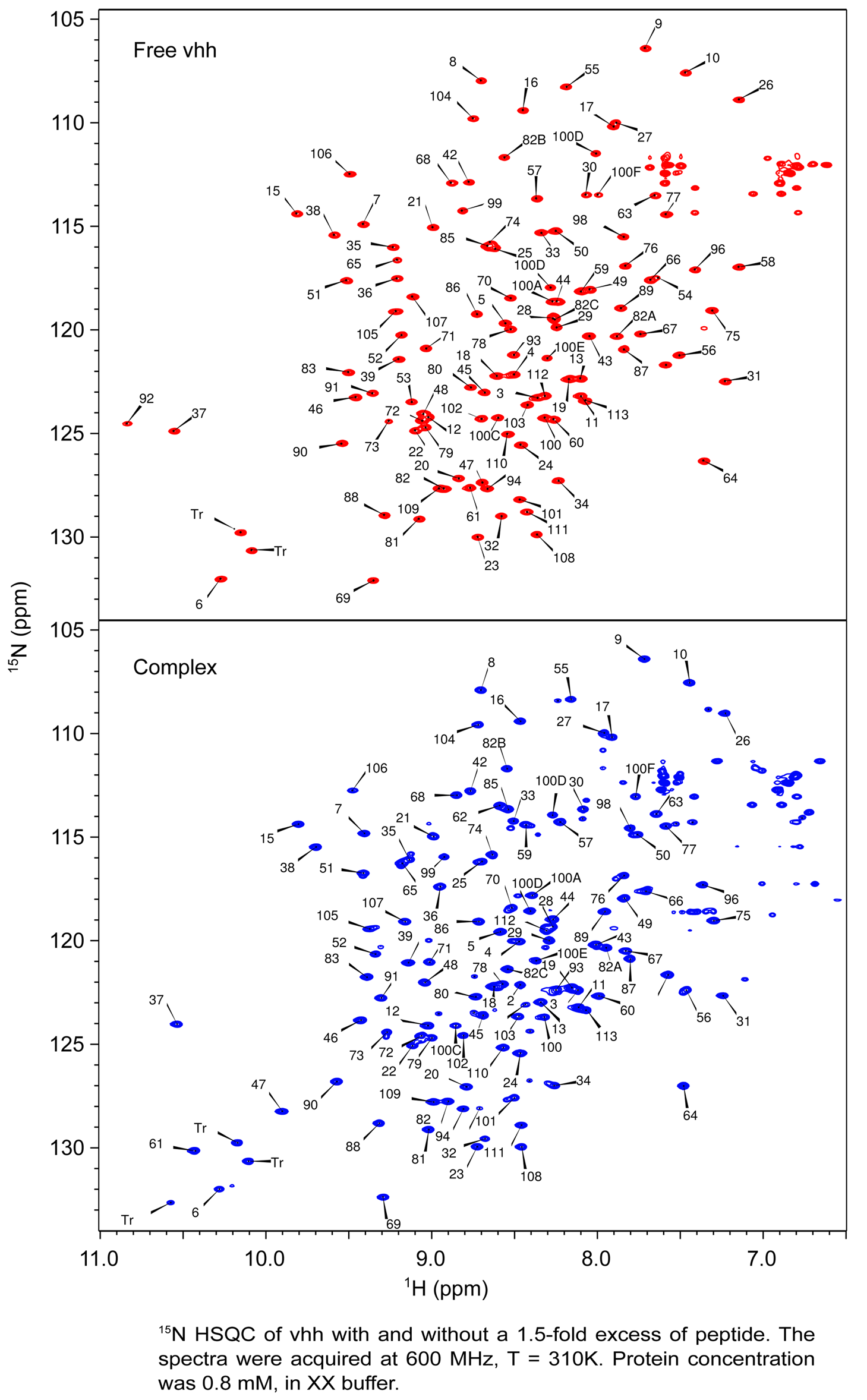

Supplement: Figure S3 — 15N HSQC of 2H10 without and with a 1.5-fold excess of peptide. The spectra were acquired at 600 MHz, T = 37 C. Protein concentration was 0.95 mM, in in 25 mM HEPES buffer and 75 mM NaCl (pH 6.7). (TIF) [file ppat.1003202.s003.tif]

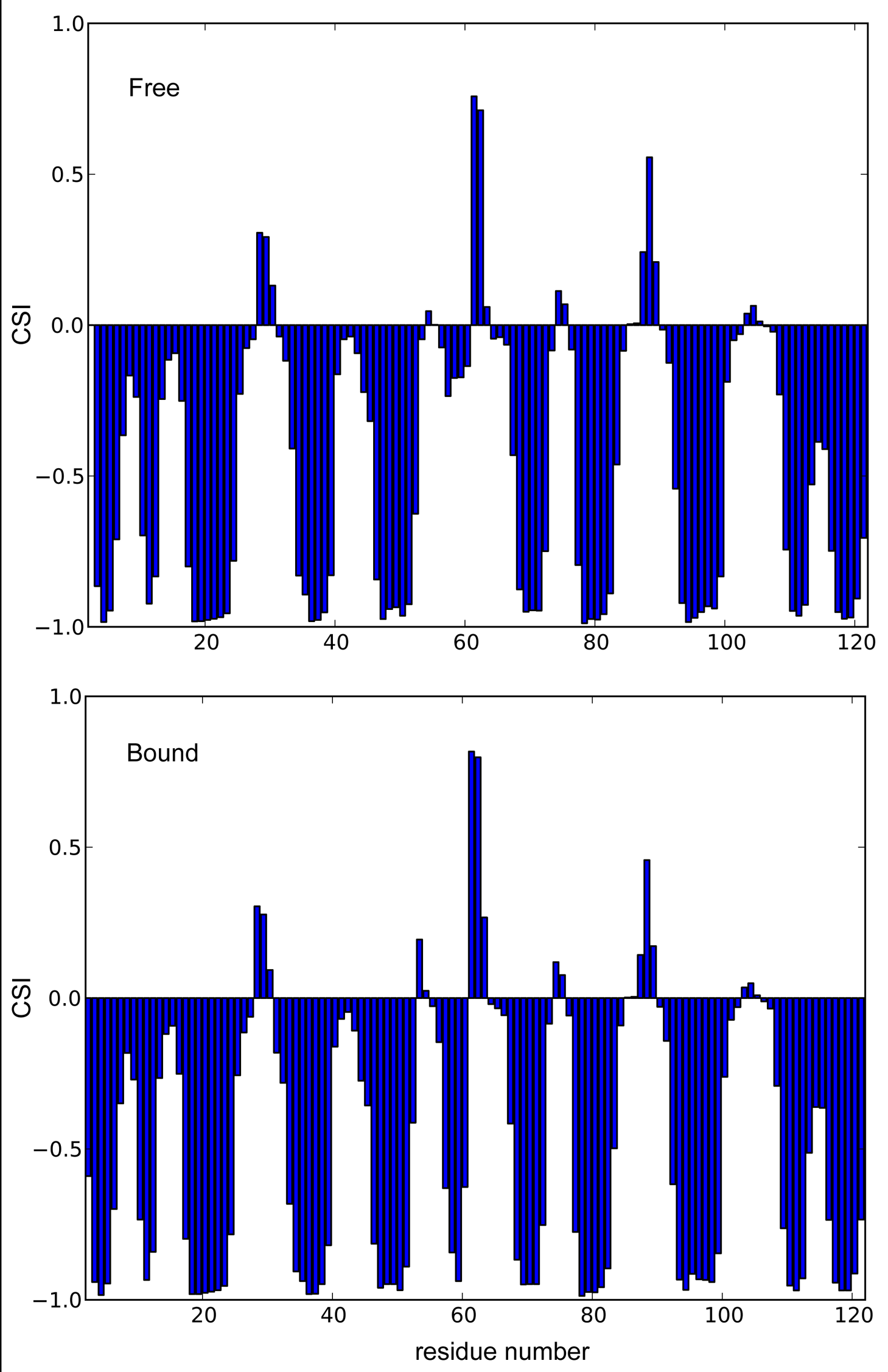

Supplement: Figure S4 — Secondary structure propensity of the free and bound form of 2H10 determined from CB, CA, CO, N chemical shift analysis using the program TALOS+. The chemical shift index (CSI) value of 1 corresponds to 100% helical conformation propensity and a value of −1 corresponds to a 100% β-strand conformation propensity. (TIF) [file ppat.1003202.s004.tif]

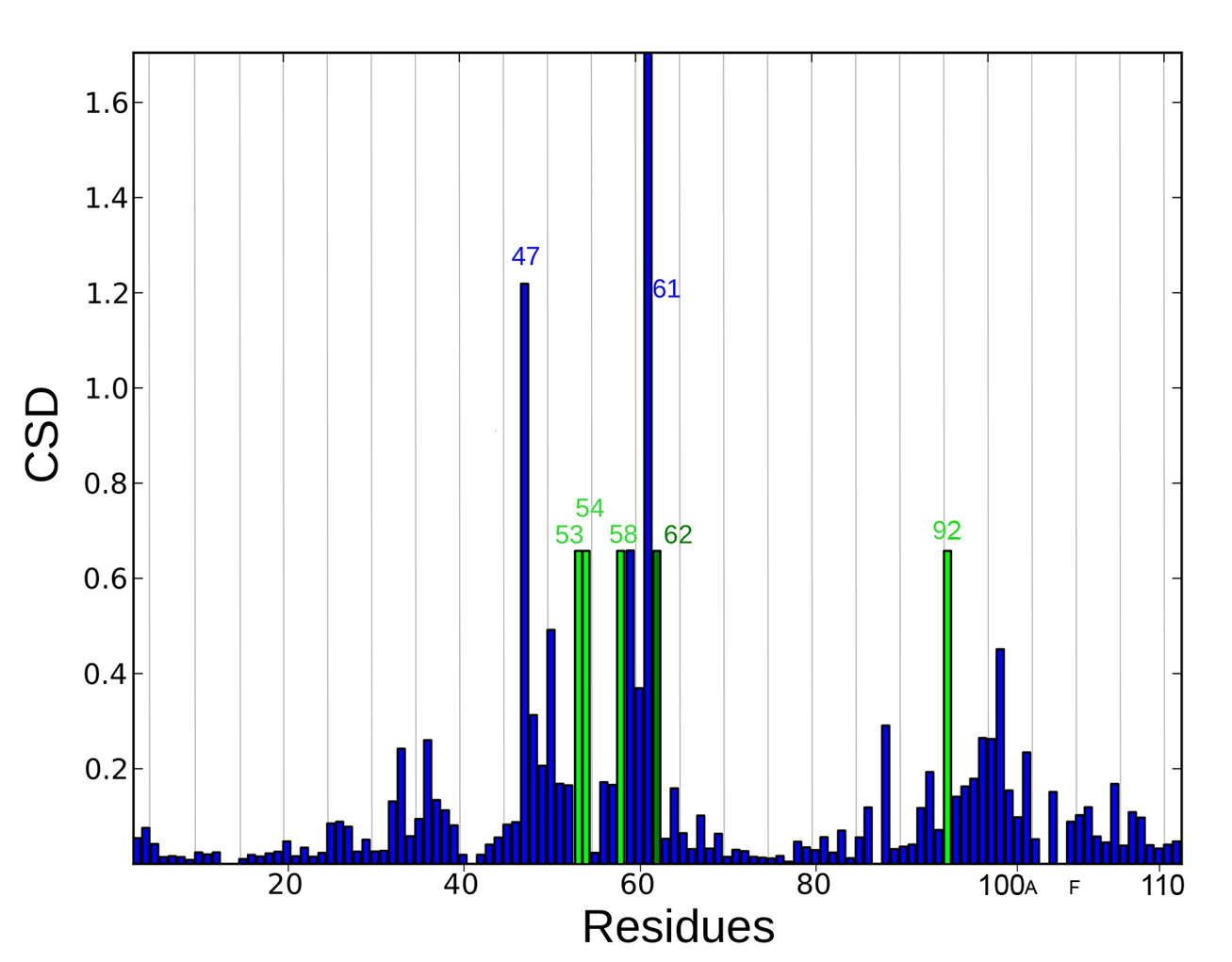

Supplement: Figure S5 — Histograms showing the value of the chemical shift difference (CSD) in 2H10 induced upon binding with the gp41 peptide (1.5 fold excess). Residue numbers are indicated on the X axis (residues for which CSD is missing are prolines. The green and pale green bars with an arbitrary height represent residues whose NH correlation is not present in the 15N-HSQC spectrum for the free and bound form of 2H10, respectively (probably because of conformation exchange broadening). The CSI analysis of the free and bound form of 2H10 show that upon addition of an excess of ligand, the secondary structure of the protein does not change. (TIF) [file ppat.1003202.s005.tif]
